# Combatting sepsis-induced myocardial dysfunction: emerging mechanisms and immunomodulatory breakthroughs

**DOI:** 10.3389/fimmu.2025.1643048

**Published:** 2025-11-03

**Authors:** Can Liu, Hanfeng Liu, Yunxing Li, Yangxi Zeng, Xinyu Wang, Yuhan Mou, Bin Liao, Juyi Wan

**Affiliations:** ^1^ Department of Cardiovascular Surgery, The Affiliated Hospital, Southwest Medical University, Metabolic Vascular Diseases Key Laboratory of Sichuan Province, Key Laboratory of Cardiovascular Remodeling and Dysfunction, Luzhou, Sichuan, China; ^2^ Key Laboratory of Medical Electrophysiology, Ministry of Education & Medical Electrophysiological Key Laboratory of Sichuan Province, (Collaborative Innovation Center for Prevention of Cardiovascular Diseases), Institute of Cardiovascular Research, Southwest Medical University, Luzhou, Sichuan, China

**Keywords:** sepsis, myocardial injury, inflammation, sepsis-induced myocardial dysfunction, targeted therapy

## Abstract

Sepsis-induced myocardial dysfunction (SIMD) critically contributes to mortality in systemic inflammatory responses, driven by multifaceted mechanisms including dysregulated inflammation, immunosuppression, oxidative stress, and autonomic dysfunction. Emerging pathways involve m6A RNA methylation (mediated by methyltransferase METTL3), which coordinates inflammation, apoptosis, and ferroptosis through transcriptomic rewiring. Extracellular vesicles (EVs) serve dual roles: propagating injury via microRNA-885-5p/HMBOX1-induced pyroptosis and delivering therapeutic cargo (e.g., microRNA-223) to suppress inflammation. Mitochondrial dysfunction, marked by reactive oxygen species (ROS)-NOD-like receptor family pyrin domain-containing 3 (NLRP3) inflammasome activation and impaired sarco/endoplasmic reticulum calcium ATPase 2a (SERCA2a) stability, exacerbates metabolic disorder. Autonomic neuromodulation strategies, such as electroacupuncture and noninvasive vagus nerve stimulation, attenuate cardiac injury by rebalancing neuroimmune interactions. Complement hyperactivation (C5a-C5a receptor axis) and immune checkpoint inhibitors (e.g., anti-programmed death-ligand 1 [PD-L1] antibodies) show preclinical efficacy. However, challenges persist in addressing immune heterogeneity, dynamic biomarker profiling, and optimal therapeutic timing. This review bridges mechanistic discoveries to clinical innovation, proposing a paradigm shift toward precision therapies. Future research must bridge mechanistic insights with clinical innovation. By harmonizing pathophysiological understanding with precision medicine approaches, this synthesis underscores the potential to transform SIMD management from supportive care to targeted functional recovery.

## 1 Introduction

Sepsis manifests as a life-threatening multiorgan dysfunction caused by a dysregulated host response to infection (1). Notably, sepsis is not a singular disease entity but rather a clinical syndrome encompassing incompletely defined pathobiological mechanisms (2). SIMD is part of systemic critical illness, and its occurrence and progression are influenced by failure of other organs and the patient’s underlying health status. This contemporary definition underscores the primacy of the host’s maladaptive response to infection, where the pathophysiological consequences surpass the direct effects of microbial invasion in driving mortality (3). Global epidemiological data indicate over 19 million sepsis cases annually, with approximately 6 million fatalities and 3 million survivors experiencing persistent cognitive impairments (4).

Myocardial injury represents one of the most lethal complications of sepsis, characterized by impaired myocardial contractility and diastolic function (5). As the predominant cause of mortality in most intensive care units (ICUs), sepsis accounts for over 250,000 deaths annually in the United States alone, with incidence rates demonstrating consistent year-over-year increases. Reported mortality reaches 38% in septic shock cases, constituting the principal fatal outcome among ICU-managed sepsis patients (6). Particularly alarming are cases demonstrating severe myocardial dysfunction with reduced cardiac index during sepsis progression, where mortality rates exceed 80% (7). Sepsis progression to severe sepsis or septic shock carries an exceptionally high mortality rate, making early recognition, prompt infection control, and timely restoration of organ perfusion critically imperative for patients. These interventions constitute critical determinants for modulating disease progression and severity. Nevertheless, the pathogenesis of sepsis-induced myocardial injury remains incompletely elucidated (5, 8).

Clinical investigations demonstrate that during sepsis development following infection, sustained and excessive inflammatory responses trigger activation of immune cells (including neutrophils and macrophages), excessive cytokine release, and complement system activation (9, 10). These cascading events culminate in immune dysregulation characterized by concurrent hyperinflammation and immunosuppression, ultimately driving tissue and organ damage (11).

The clinical evaluation of SIMD relies on the integration of multiple parameters, including cardiac ultrasound indicators (such as left ventricular ejection fraction LVEF, global longitudinal strain GLS) and biomarkers (such as troponin, BNP). However, the interpretation of these tools in the context of sepsis is often complicated by limitations such as load dependency and non-specificity (12).

Despite decades of research, the high mortality of SIMD persists, underscoring critical gaps in understanding its evolving pathobiology. While existing reviews have catalogued classical pathways (e.g., cytokine storms, mitochondrial dysfunction), emerging mechanisms—such as the STING-cGAS axis-driven mtDNA sensing, m6A methylation-mediated transcriptomic rewiring, and EVs acting as both pathogenic vectors and therapeutic vehicles—remain underappreciated in current syntheses (13). Furthermore, innovative interventions like autonomic neuromodulation and EV-based therapies challenge conventional symptom-centric paradigms, yet their integration into a unified framework is lacking. This review uniquely bridges these gaps by:

1. Systematically dissecting newly identified pathways, including neuroimmune crosstalk and EV-mediated organelle stress propagation;
2. Reconceptualizing EVs as dual agents—highlighting their role in exacerbating cardiomyocyte pyroptosis via miR-885-5p/HMBOX1 signaling while showcasing engineered EVs delivering miR-223 to reduce mortality in preclinical models;
3. Critically appraising clinical advances, such as ongoing clinical trials of anti-PD-L1 antibodies and vagus nerve stimulation devices, which may collectively expand therapeutic windows from “hemodynamic rescue” to “mechanism-targeted repair”.

By synthesizing fragmented discoveries into actionable insights, this work provides a roadmap for transitioning SIMD management from reactive support to precision medicine.

## 2 The pathogenetic mechanisms of myocardial injury in sepsis

### 2.1 Inflammatory Response

**Figure 1:**
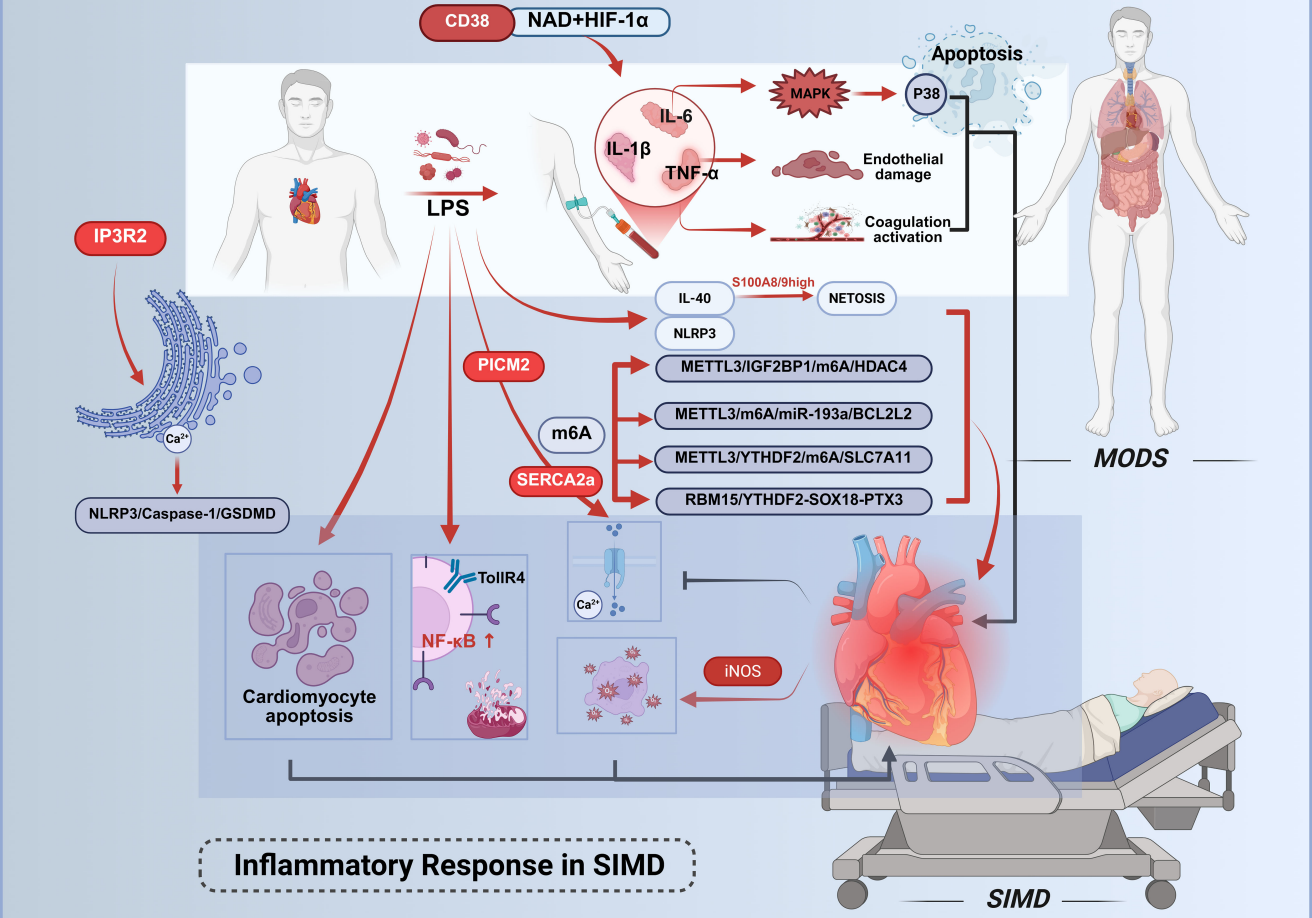
Inflammatory mechanisms in septic cardiac injury. Cardiac injury in sepsis is driven by inflammatory cascades initiated through Gram-negative bacterial LPS. LPS directly activates pro-inflammatory cells or binds to TLR4, triggering pathways such as NF-κB to upregulate the expression of TNF-α, IL-1β, and IL-6. TNF-α mediates primary inflammatory manifestations, while IL-6 selectively activates the p38 MAPK signaling pathway. Elevated IL-6 levels promote coagulation cascade activation and vascular endothelial dysfunction, thereby suppressing myocardial contractility. IL-40 drives sepsis progression. CD38high monocytes predict 28-day mortality in sepsis; their CD38-NAD^+^-HIF-1α axis drives glycolysis.Concurrently, LPS activates the NLRP3 inflammasome, accelerating multiorgan dysfunction.Central to myocardial injury regulation is m6A RNA methylation. The methyltransferase METTL3 coordinates multiple pathological processes through distinct downstream pathways, collectively exacerbating myocardial dysfunction in SIMD. This figure was created using BioRender (biorender.com).

The progression of sepsis-induced cardiac dysfunction involves multiple mechanistic pathways. (**Figure 1**) Bacterial infection serves as the primary etiology of sepsis, with LPS – a dominant component of Gram-negative bacterial membranes – playing an indispensable role in sepsis-associated cardiac injury. During sepsis, LPS stimulation elevates the expression and release of proinflammatory cytokines, including IL-6 and TNF-α. These inflammatory mediators recruit additional immune cells to cardiac tissue, which subsequently amplifies the intracardiac inflammatory cascade (14). Recent studies associated with murine models and observational clinical studies highlight interleukin-40 (IL-40) as a novel mediator exacerbating sepsis pathogenesis (15). Elevated IL-40 levels correlate with disease severity and early mortality in both adult and pediatric septic patients. Mechanistically, IL-40 promotes neutrophil influx and S100A8/9^high^ neutrophil activation, driving neutrophil extracellular trap (NET) formation (NETosis), which amplifies systemic inflammation and organ dysfunction. Genetic knockout of IL-40 in murine models attenuates cytokine storms, reduces NETosis-related markers (MPO/dsDNA), and improves survival, positioning IL-40 as a potential therapeutic target to mitigate hyperinflammation. Sepsis has long been recognized as a cytokine storm syndrome. This condition is pathologically characterized by the acute release of multiple inflammatory mediators, including TNF-α, IL-6, and IL-1β. The excessive liberation of these inflammatory factors induces direct tissue and organ damage, while accumulating evidence has demonstrated an association between such inflammatory responses and dysregulated cellular death pathways (16–18). Elevated levels of potent cytokines, including IL-6, IL-1β, and TNF-α, are detectable in patients diagnosed with early-stage sepsis. These promising results from preclinical and preliminary clinical observations still require further validation in larger cohorts and through experimental intervention studies to clarify their clinical translational potential, limitations, and the need for additional verification.

Activation of immune cells serves as a prerequisite for inflammatory responses, while their subsequent immune responses are considered significant contributors to the development of SIMD (13). Following pathogenic challenge by microbial components such as LPS, this endotoxin engages Toll-like receptor 4 (TLR4) to initiate inflammatory mediator production. TLR4 activation upon LPS stimulation triggers downstream signaling cascades, particularly the nuclear factor kappa-B (NF-κB) pathway (19, 20). NF-κB governs the transcriptional regulation of numerous pro-inflammatory genes, subsequently inducing DNA damage and mitochondrial dysfunction, thereby exacerbating the progression of cardiac failure (21–25). Clinical investigations reveal marked elevations in inflammatory cytokines (IL-6, IL-1β, TNF-α) within plasma and target organs, including myocardial tissue. Localized TNF-α elevation manifests cardinal inflammatory symptoms - pyrexia, edema, algogenesis, and erythema. This pleiotropic cytokine mediates tissue damage through multiple mechanisms: depression of cardiac output, induction of microvascular thrombosis, and mediation of systemic capillary leak syndrome. TNF-α amplifies and prolongs inflammatory responses by activating secondary cytokine release (IL-1, HMGB1) and stimulating production of eicosanoids, nitric oxide, and reactive oxygen species (26). While TNF-α is indispensable for complete inflammatory expression during pathogen invasion, self-limiting inflammation typically correlates with diminished TNF activity. IL-6 demonstrates dual pathological effects through its engagement of the p38 mitogen-activated protein kinase (MAPK) signaling pathway, recently implicated in apoptotic regulation. Elevated IL-6 concentrations activate coagulation cascades and vascular endothelium while suppressing myocardial contractility (27). Both IL-6 and TNF-α exert cardiodepressant effects via sphingomyelinase pathway activation in cardiomyocytes, impairing calcium homeostasis and inducing pathological overexpression of inducible nitric oxide synthase (iNOS) (28).

Dysregulation of calcium homeostasis constitutes a pivotal mechanism underlying SIMD, orchestrated by the interplay of multiple molecular pathways. Primarily, pyruvate kinase M2 (PKM2) safeguards calcium ion reuptake and excitation-contraction coupling through direct interaction with SERCA2a, thereby maintaining its protein stability. Experimental evidence demonstrates that PKM2 deficiency markedly reduces SERCA2a expression, resulting in delayed calcium transient decay, impaired contractile function, and exacerbated cardiomyocyte apoptosis, whereas PKM2 overexpression or pharmacological activation via TEPP-46 rescues these pathological alterations (29). Secondly, inositol 1,4,5-trisphosphate receptor type 2 (IP3R2)-mediated endoplasmic reticulum calcium efflux activates the NOD-like receptor family pyrin domain-containing 3 (NLRP3)/Caspase-1/GSDMD axis, driving pyroptotic cardiomyocyte death. Notably, IP3R2 forms a self-amplifying positive feedback loop with endoplasmic reticulum stress markers (e.g., upregulated ATF4 and CHOP), intensifying calcium signaling dysregulation and inflammatory injury (30). Furthermore, post-translational modifications of SERCA2a, exemplified by succinylation at lysine 352 (K352), directly impair calcium cycling by accelerating its ubiquitination and proteasomal degradation. Conversely, SIRT2-mediated desuccinylation restores SERCA2a stability and enzymatic activity, ameliorating cardiac functional parameters in septic models (31). Collectively, the PKM2-SERCA2a axis, IP3R2/calcium-NLRP3 pathway, and SERCA2a succinylation modification constitute a multi-layered regulatory network governing calcium homeostasis disruption, delineating critical molecular targets and therapeutic strategies for SIMD intervention.

Emerging evidence implicates NLRP3 inflammasome activation in sepsis-associated multiorgan dysfunction. Experimental models demonstrate that NLRP3 inhibition significantly attenuates LPS-induced acute lung injury (32) and improves sepsis-related inflammatory responses and organ damage (33). Notably, targeted modulation of NLRP3 inflammasome activity has been proposed as a potential therapeutic strategy against LPS-mediated cardiac injury (34, 35).

Recent advances in understanding the molecular pathogenesis of SIMD have highlighted the central role of m6A RNA methylation in regulating myocardial injury. Accumulating evidence demonstrates that the methyltransferase METTL3 orchestrates diverse pathological processes—including inflammation, apoptosis, and ferroptosis—through distinct downstream pathways: 1) METTL3 catalyzes m6A modification at the 3’-UTR of HDAC4 mRNA, which is recognized by the reader protein IGF2BP1 to enhance its stability, resulting in elevated HDAC4 expression that exacerbates pro-inflammatory cytokine release (e.g., IL-6, TNF-α) and apoptotic cell death (36); 2) METTL3 facilitates the maturation of pri-miR-193a via m6A modification, with the mature miR-193a directly suppressing the anti-apoptotic gene *BCL2L2* by targeting its 3’-UTR, thereby activating the caspase-3-dependent apoptotic cascade and amplifying inflammatory responses (37); 3) METTL3-mediated m6A modification promotes YTHDF2-dependent degradation of *SLC7A11* mRNA, inhibiting cystine uptake and glutathione synthesis, which culminates in lipid ROS accumulation and ferroptotic cell death (38). Additionally, an independent regulatory axis involving RBM15/YTHDF2-SOX18-PTX3 has been identified, wherein m6A modification destabilizes *SOX18* mRNA, ablating its transcriptional repression of PTX3 and subsequently driving NLRP3 inflammasome assembly and cardiomyocyte pyroptosis (39). Collectively, these findings underscore that m6A-dependent epigenetic regulation orchestrates a multifaceted molecular network, coordinating inflammation, apoptosis, ferroptosis, and pyroptosis to exacerbate myocardial dysfunction in SIMD. This mechanistic complexity highlights the therapeutic potential of targeting m6A-modifying enzymes or their downstream effectors for multi-pathway intervention strategies.

Sepsis compromises immune cell functionality both directly and indirectly via damage-associated molecular patterns (DAMPs), including proinflammatory cytokines (IL-1β, TNF-α, IL-6) (40, 41), iNOS (42, 43), reactive oxygen species (ROS) (44), heat shock proteins, HMGB1, histones, activated complement components, and mitochondrial DNA. The excessive inflammatory response has been identified as a principal underlying mechanism in this pathological cascade (45–47). A recent study demonstrated that circulating CD38^high^ monocytes exhibit time-dependent expansion within 24 hours of sepsis onset, with their abundance independently correlating with 28-day mortality in bacterial sepsis patients (48). Mechanistically, these cells drive immune-metabolic dysregulation via the CD38-NAD^+^-HIF-1α axis, triggering hyperactivation of glycolysis (evidenced by elevated extracellular acidification rates and upregulated GLUT1/GAPDH expression). This glycolytic shift generates methylglyoxal (MGO), which reinforces CD38 expression through STAT1 phosphorylation, establishing a pathogenic feedforward loop. In experimental models, genetic or pharmacological inhibition of CD38 attenuated pro-inflammatory cytokine production (e.g., IL-6, CXCL1), improved survival in cecal ligation and puncture (CLP)-induced sepsis, and mitigated multi-organ dysfunction. These findings delineate CD38^high^ monocytes as pivotal orchestrators of sepsis-associated immunopathology through a self-amplifying metabolic-inflammatory circuitry.

### 2.2 Immunosuppression

**Figure 2:**
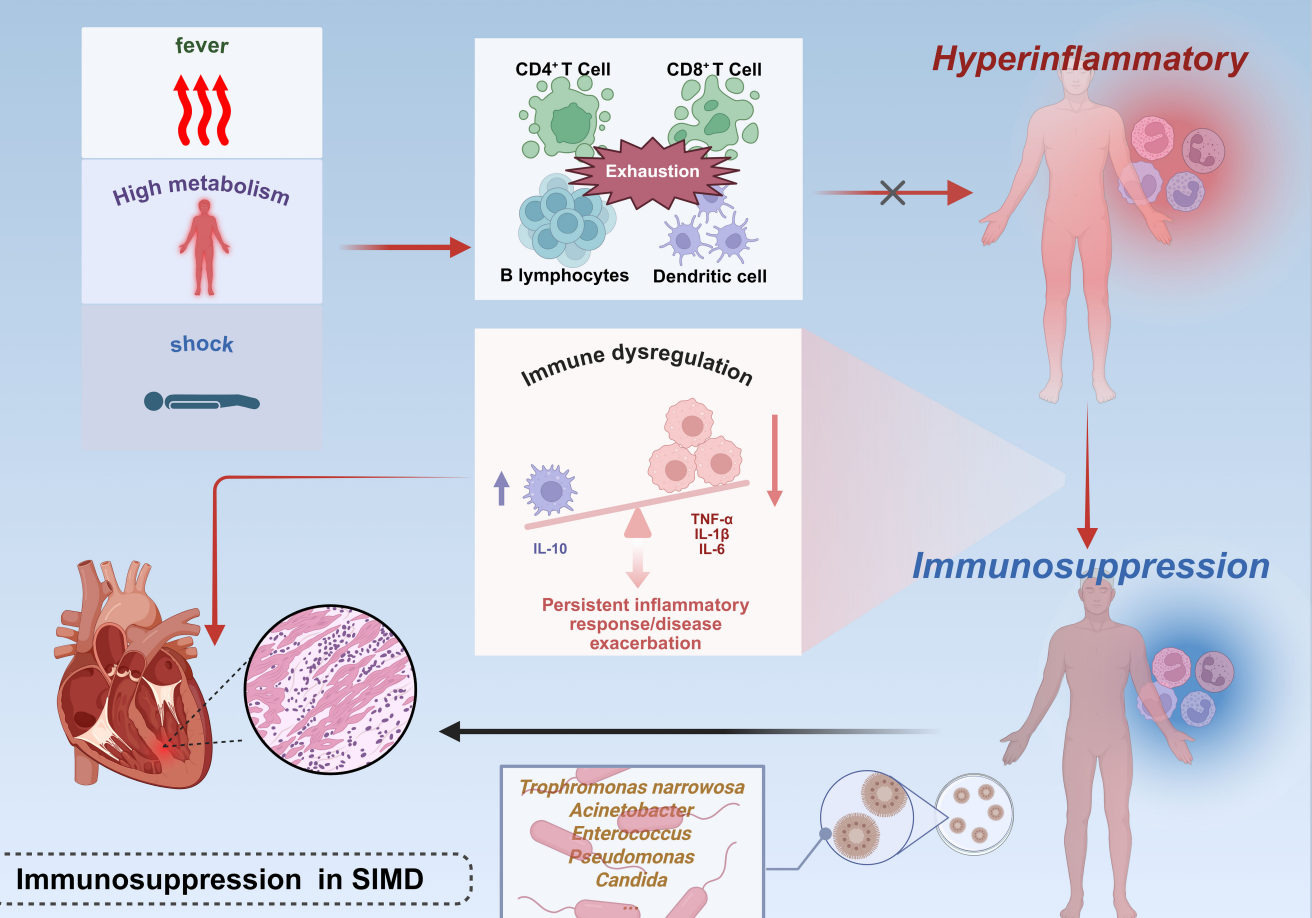
Immunosuppressive mechanisms in septic cardiac injury.The early phase of sepsis is characterized by a hyperinflammatory response marked by a surge in pro-inflammatory cytokines, concurrent with functional exhaustion of CD4^+^/CD8^+^ T cells, B cells, and follicular DCs, which compromises anti-inflammatory capacity. Monocyte dysfunction (evidenced by impaired pro-inflammatory cytokine production) and lymphocyte abnormalities (including reduced proliferative activity and aberrant anti-inflammatory cytokine elevation, such as IL-10) collectively drive a pro-/anti-inflammatory imbalance. This immunosuppressive state predisposes to secondary infections, further amplifying systemic inflammatory responses and exacerbating myocardial injury. This figure was created using BioRender (biorender.com).

The repeated failure of clinical trials targeting inflammatory mediator blockade or pathogen recognition pathways in sepsis has substantiated the hypothesis that disease progression extends beyond hyperinflammation alone (49, 50). (**Figure 2**) During the progression of shock, the immune response undergoes dynamic evolution: initially presenting as SIRS (systemic inflammatory response syndrome), characterized by massive release of pro-inflammatory cytokines and widespread activation of immune cells, which aims to initiate repair but can easily lead to tissue damage; followed by CARS (compensatory anti-inflammatory response syndrome), which suppresses inflammation through anti-inflammatory mediators and upregulation of immune checkpoints to restore homeostasis, but excessive suppression may result in immune disorder and increased risk of infection; the interplay of these two states forms MARS (mixed antagonist response syndrome), manifested as immune imbalance and functional dysregulation, ultimately progressing to multiple organ dysfunction and systemic failure. These three stages constitute the core continuum of immunopathology in shock, determining the direction of clinical outcomes (51). During the early phase of sepsis, the host mounts an immune response to infection or injury, characterized by a hyperinflammatory phenotype manifesting as shock, pyrexia, and a hypermetabolic state. Concurrently, progressive immune cell exhaustion—marked by depletion of CD4^+^ T lymphocytes, CD8^+^ T lymphocytes, B lymphocytes, and follicular dendritic cells (DCs)—compromises the capacity to counteract overwhelming inflammation, thereby exacerbating disease severity (52, 53). Sepsis-induced immunosuppression exhibits two hallmark features: impaired monocytic production of proinflammatory cytokines (particularly TNF-α) upon endotoxin challenge and diminished lymphocyte proliferative capacity. Paradoxically, this occurs despite the theoretical expectation of clonal lymphocyte expansion during immune activation (54). Clinical investigations reveal elevated plasma concentrations of both proinflammatory cytokines and the potent anti-inflammatory cytokine IL-10 in septic patients (55). Notably, severe sepsis frequently presents with concurrent persistent inflammation and immunosuppression—a state termed “ Immune disorder “ (56) van Dissel et al. analyzed cytokine profiles in 464 patients, demonstrating that elevated IL-10/TNF-α ratios correlate with mortality in community-acquired infections (57). Microbiological evidence further supports impaired host immunity in sepsis. Common nosocomial pathogens in ICU settings—including *Stenotrophomonas maltophilia*, *Acinetobacter* spp., *Enterococcus* spp., *Pseudomonas aeruginosa*, and *Candida* spp.—are frequently low-virulence or opportunistic organisms, serving as biomarkers of profound immunosuppression (58, 59).(**Table 1**) This immunosuppressive milieu perpetuates inflammatory persistence and drives clinical deterioration through multiple mechanisms: secondary infections amplify systemic inflammation, while inflammation-mediated pathologies exacerbate organ dysfunction, culminating in cardiac failure and mortality. Postmortem analyses of sepsis fatalities reveal marked immunosuppression, characterized by reduced cytokine production (via upregulation of inhibitory receptors on organ-infiltrating T cells) in splenic and pulmonary immune cells (60). Notably, sustained infection per se does not fully account for sepsis-related mortality (61). Experimental evidence establishes a causal link between immune effector cell loss and outcomes. Multiple independent studies demonstrate that anti-apoptotic therapies preserve immune effector populations and improve survival in clinically relevant sepsis models (62–64). The impact of sepsis on the heart extends far beyond the acute phase, as the myocardial dysfunction it induces persists even after patients are discharged from the hospital and significantly increases long-term cardiovascular risk. This process begins with myocardial damage caused by the cytokine storm and myocardial depressant factors during the acute phase, while the long-term effects stem from persistent immune-inflammatory disturbances and accelerated vascular aging mechanisms. The latter, through oxidative stress and aging signaling pathways such as p53/p21 and p16, promotes endothelial dysfunction and the progression of atherosclerosis, ultimately leading to a significant increase in the risk of major adverse cardiovascular events (such as heart failure, myocardial infarction, and stroke) in survivors. Therefore, the cardiac damage caused by sepsis is actually a continuous pathological process that extends from acute injury to chronic cardiovascular disease, and requires long-term monitoring and intervention (65). Despite these advances, the precise mechanisms underlying immunosuppression in SIMD remain incompletely elucidated.

**Table 1:** Septic myocardial disease is mainly induced by infections from the following pathogens, predominantly bacteria, with occasional occurrences of fungi or viruses.

Pathogenetic basis of sepsis-induced myocardial injury		
Pathogen category	Common pathogens	Mechanisms
Gram-negative bacteria (50%~60%)	*Escherichia coli* *Klebsiella pneumoniae* *Pseudomonas aeruginosa* *Acinetobacter baumannii*	Endotoxin (LPS) leads to myocardial suppression by activating an inflammatory response.
Gram-positive bacteria (30%~40%)	*Staphylococcus aureus* *Streptococcus pneumoniae* *Enterococcus*	Exotoxins (such as superantigen TSST-1) directly damage myocardial cells.
Fungi (5%~10%) Viruses (less common)	*Candida* spp. *Influenza viruses* (e.g., H1N1) *Enteroviruses* (e.g., coxsackievirus)	Fungal invasion leads to microcirculation disturbances and amplification of inflammation. Direct myocardial cell lysis or immune-mediated injury.

### 2.3 Oxidative stress

Systemic ischemic multiorgan dysfunction is the concentrated manifestation of systemic ischemia/reperfusion injury under shock conditions. Ischemia/reperfusion injury is accompanied by severe oxidative stress, leading to an outburst of reactive oxygen/nitrogen species and triggering cellular damage (51). The core mechanisms of I/R highly overlap with the pathophysiological process of SIMD, forming a self-amplifying vicious cycle. The pathophysiological process of SIMD is jointly driven by I/R injury at three levels: cellular (particularly mitochondrial), microcirculatory, and systemic immune. In the early stage of shock, cells initiate metabolic reprogramming (such as enhanced glycolysis) via pathways like HIF-1α, attempting to maintain themselves before a critical physiological threshold known as the “safe return point.” Within this window, if perfusion and oxygen supply can be promptly restored, mitochondrial function remains reversible, and cells can survive (66). However, persistent hypoperfusion and acidosis disrupt this balance. Once injury surpasses the “safe return point,” mitochondria undergo irreversible collapse, manifested as uncoupling of the electron transport chain, failure of ATP synthesis, and burst production of ROS. The hallmark of mitochondrial collapse is the sustained opening of the mitochondrial permeability transition pore (mPTP) (51). mPTP is directly triggered by calcium overload, oxidative stress, and ATP depletion during I/R. Its opening leads to the collapse of the mitochondrial membrane potential, swelling, and rupture of the outer membrane, releasing pro-apoptotic factors such as cytochrome C, directly initiating apoptosis or necrosis (67). Therefore, mPTP is regarded as the core hub connecting I/R injury to cell death, and it is also the direct execution mechanism for the massive loss of parenchymal cells such as cardiomyocytes, renal tubular epithelial cells, and neurons in SIMD. Even if macroscopic blood flow is restored, the “no-reflow” phenomenon may still occur at the microcirculatory level (51, 68). This is caused by endothelial cell swelling, leukocyte plugging, microthrombus formation, and vasoconstriction, which collectively obstruct microvascular patency. This directly results in persistent ischemia at the tissue level, hindering the delivery of oxygen and nutrients and the clearance of metabolic waste, thereby exacerbating the progression of multiple organ dysfunction syndrome. This phenomenon is the core link through which ischemia/reperfusion injury drives multiorgan dysfunction at the microcirculatory level. The “safe return point” defines the golden window for intervention, while the opening of mPTP marks the cell’s transition toward an irreversible terminal stage. Therefore, future protective strategies against shock and SIMD hold significant potential in focusing on inhibiting mPTP opening through pharmacological agents (such as cyclosporine A analogs), thereby extending the time limit of the “safe return point” and creating valuable opportunities for fundamental treatment (51).

**Figure 3:**
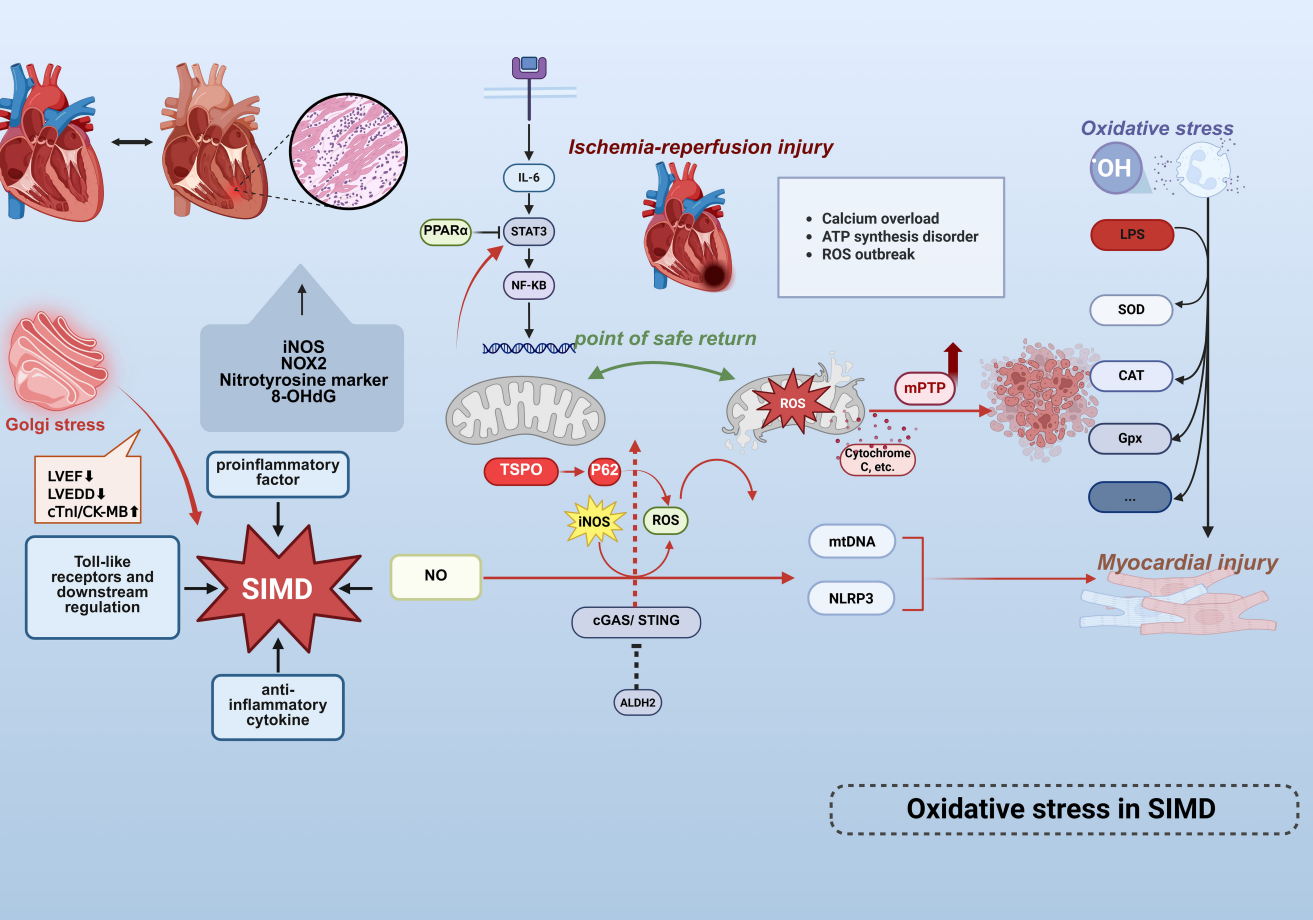
Oxidative stress mechanisms in septic cardiac injury. Cardiac tissue from non-surviving septic patients exhibits marked upregulation of iNOS, NOX2, nitrotyrosine, and 8-OHdG, highlighting the central role of OS/NS in myocardial injury.Golgi stress (inflammation/apoptosis) exacerbates sepsis-induced myocardial dysfunction (SIMD) characterized by LVEF reduction, LVEDD elevation, and cTnI/CK-MB elevation. Infiltrating leukocytes excessively release free radicals, directly damaging cardiomyocytes.LPS-induced overproduction of ROS activates the NLRP3 inflammasome, mediating cardiomyocyte death and exacerbating injury. Concurrently, ROS disrupt mitochondrial membrane potential, triggering mtDNA release as DAMPs that further amplifies inflammatory cascades. This sustained ROS generation activates the IL-6/STAT3/NF-κB signaling pathway, propagating inflammatory responses.The endogenous antioxidant defense system is compromised via reduced expression of SOD,CAT, and GPx. Conversely, mitochondrial ALDH2 mitigates LPS-induced cardiac dysfunction, inflammation, and apoptosis by suppressing the cGAS/STING pathway. SIMD is driven by ischemia/reperfusion injury, involving oxidative stress, mitochondrial dysfunction (mPTP opening), and microcirculatory disturbances. The safe return point defines the intervention window, and mPTP inhibition may provide protection.These findings collectively establish oxidative stress and mitochondrial dysfunction as core mechanisms driving cardiac involvement in sepsis. This figure was created using BioRender (biorender.com).

Comparative analyses of cardiac specimens from sepsis non-survivors versus controls have demonstrated elevated expression of iNOS, NOX2, nitrotyrosine markers, and significantly increased 8-OHdG levels, underscoring the pivotal role of oxidative stress in sepsis pathogenesis (69). (**Figure 3**) Infiltrating leukocytes exacerbate cardiac oxidative damage through excessive free radical production. Lipopolysaccharide challenge further reduces the expression of key antioxidant enzymes—including superoxide dismutase (SOD), catalase (CAT), and glutathione peroxidase (GPx)—thereby disrupting endogenous cardiac antioxidant defenses. Aldehyde dehydrogenase 2 (ALDH2), a mitochondrial-localized enzyme with established cardioprotective roles in cardiovascular diseases (CVDs), exhibits activity inversely correlated with myocardial infarction size (70).

Mitochondrial dysfunction constitutes a central pathological mechanism in SIMD, characterized by metabolic derangements, dynamics imbalance, and impaired quality control mechanisms. Emerging evidence indicates significant downregulation of peroxisome proliferator-activated receptor α (PPARα) in septic models, which suppresses critical fatty acid oxidation genes (e.g., Cpt1b, Acox1), culminating in reduced ATP synthesis, diminished activities of mitochondrial complexes I/IV, and ultrastructural cristae disruption. Concurrent dysregulation of mitochondrial fission/fusion dynamics (evidenced by aberrant DRP1/MFN1 expression) precipitates excessive PINK1/Parkin-mediated mitophagy and mitochondrial-dependent apoptosis via cytochrome c release and BAX/BCL-2 axis dysregulation (14). Notably, elevated expression of mitochondrial membrane protein TSPO during sepsis exacerbates autophagosome accumulation through p62-dependent autophagic flux inhibition, while activating the RIP1/RIP3 pathway to promote exosomal release of mitochondrial debris. This process triggers IL-6/STAT3/NF-κB inflammatory cascades, thereby amplifying myocardial injury (71). Furthermore, sepsis-induced compensatory upregulation of TMBIM1 in cardiomyocytes paradoxically fails to mitigate pathology upon its deficiency, which aggravates mitochondrial ROS accumulation, collapses membrane potential, and impedes Parkin translocation to damaged mitochondria. Such impairment in dysfunctional mitochondrial clearance exacerbates oxidative stress, inflammatory cytokine release, and contractile deterioration (72). Collectively, these mechanisms delineate mitochondrial metabolic dysregulation, autophagic imbalance, and the production of mitochondrial ROS is both a result of inflammatory damage and an amplifier of it. Recent investigations have further revealed that ALDH2 activation mitigates oxidative stress and inflammation, counteracting hyperglycemia-induced myocardial fibrosis and apoptosis (73). Mechanistically, ALDH2 confers protection against SIMD by attenuating LPS-triggered cardiac impairment, inflammation, and apoptosis through suppression of the cGAS/STING pathway (74).

Emerging evidence implicates dysregulated nitric oxide (NO) release and iNOS-driven metabolic perturbations in SIMD pathogenesis, alongside disproportionate pro-/anti-inflammatory cytokine expression and aberrant Toll-like receptor signaling (75, 76). Previous investigations have established that LPS-induced ROS overproduction exacerbates hyperglycemic and hypoxia/reoxygenation-mediated myocardial injury via NLRP3 inflammasome-dependent cardiomyocyte death (77). Free radicals exert dual detrimental effects: disrupting mitochondrial membrane potential to amplify organellar damage, and inducing mitochondrial DNA (mtDNA) release—which functions as intracellular DAMPs to exacerbate inflammatory responses, thereby establishing a self-perpetuating pathological cycle. Mitochondria have been identified as primary targets during acute septic injury (12), with their dysfunction driving ROS accumulation and subsequent IL-6/STAT3/NF-κB pathway hyperactivation (14). Notably, iNOS remains physiologically inactive under normal conditions but becomes pathologically upregulated during sepsis. Excessive NO generation correlates with hemodynamic alterations—including arterial hypotension and increased cardiac workload—that predispose to septic cardiomyopathy. These findings collectively support the hypothesis that oxidative/nitrosative stress (OS/NS) constitutes a central mechanistic determinant of cardiac involvement in sepsis-related mortality. Golgi stress exacerbates SIMD (LVEF↓, LVEDD↑, cTnI/CK-MB↑) via inflammation and apoptosis, validated by aggravated injury with BFA (Golgi agonist) and rescue with GSH (inhibitor), highlighting Golgi integrity as a therapeutic target for SIMD (78).

### 2.4 Autonomic dysregulation

**Figure 4:**
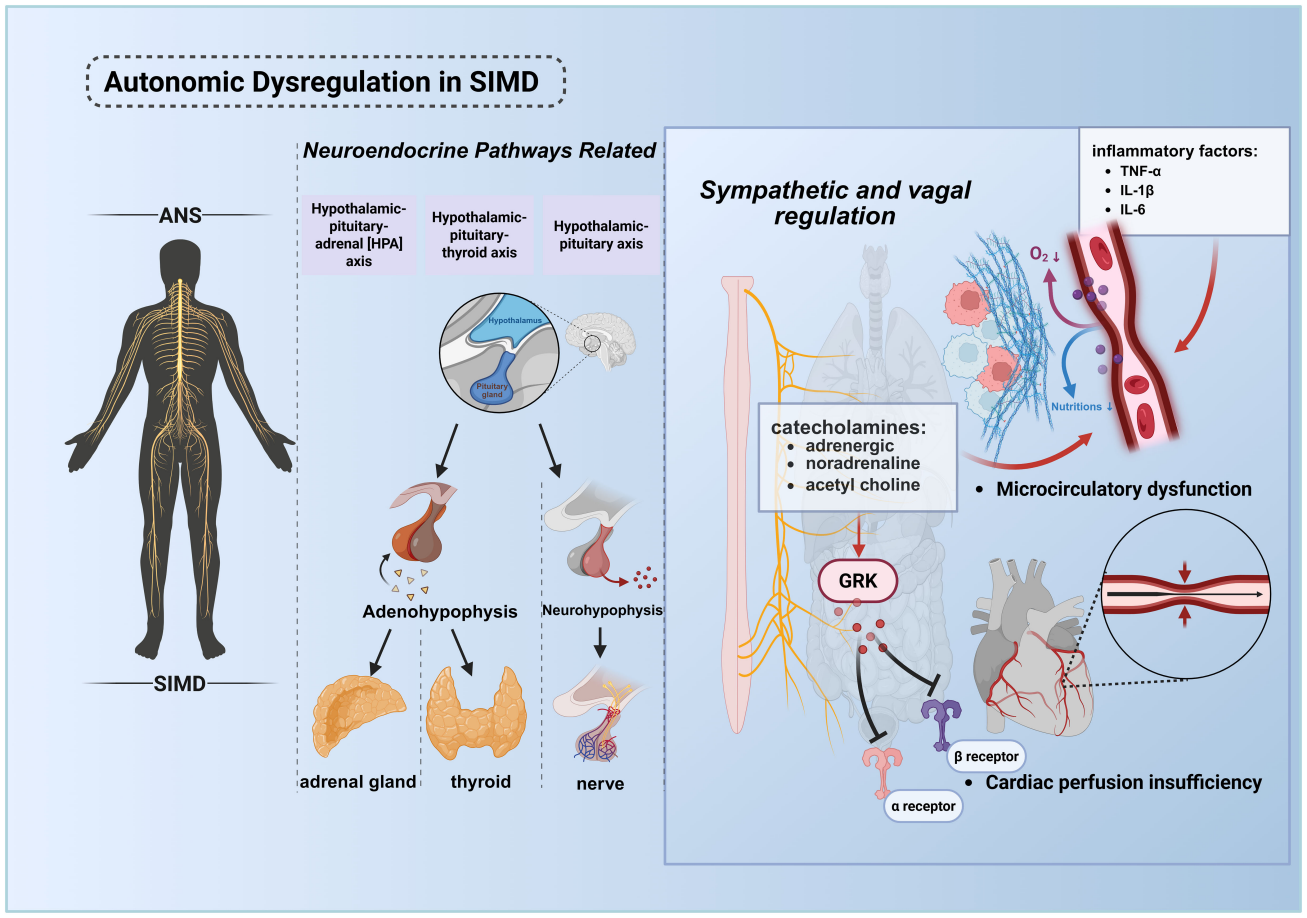
Pathological mechanisms of ANS dysregulation in septic cardiac injury. The ANS maintains systemic homeostasis by regulating cardiac output, vascular tone, inflammatory responses, immune equilibrium, and energy metabolism. During sepsis, infection or tissue injury activates the central nervous system, triggering the HPA axis, hypothalamic-pituitary-thyroid axis, and hypothalamic-neurohypophysial axis to orchestrate stress responses. ANS dysregulation, coupled with elevated inflammatory mediators (e.g., cytokine storm), synergistically promotes microcirculatory disturbances, culminating in multiorgan failure, including cardiac dysfunction. In septic shock, desensitization of adrenergic receptors leads to myocardial depression and vascular paralysis, exacerbating the vicious cycle and increasing mortality. This figure was created using BioRender (biorender.com). ANS, The autonomic nervous system.

The autonomic nervous system (ANS), a master regulator orchestrating physiological functions across virtually all organ systems (79), plays a critical role in sepsis-associated pathophysiology. (**Figure 4**) Homeostatic processes governed by the ANS—particularly those relevant to sepsis progression—include cardiac output modulation, vascular tone regulation, respiratory control, inflammatory response modulation, adaptive immune regulation, gastrointestinal motility, and thermoregulation (80–82). Clinical evidence indicates that ANS dysregulation in response to infection is both prevalent in septic patients and serves as an early biomarker of incipient organ dysfunction (79). This pathophysiological nexus is intrinsically linked to neuroendocrine physiology, as autonomic and neuroendocrine pathways (e.g., hypothalamic-pituitary-adrenal [HPA] axis, hypothalamic-pituitary-thyroid axis, hypothalamic-neurohypophysis axis) become co-activated during systemic perturbations such as inflammation and tissue injury (83).

Pathogen and damage sensing occurs through dual mechanisms: cytokine-mediated central activation and ANS-afferent fiber signaling via receptors for DAMPs and pathogen-associated molecular patterns (PAMPs) (84, 85). In sepsis, pathological hyperactivation of both sympathetic and parasympathetic ANS branches drives excessive release of catecholamines (predominantly epinephrine and norepinephrine) and acetylcholine, inducing vasoconstriction, elevated peripheral resistance, and heightened tissue energy demand. This maladaptive response, arising not only from inflammatory cytokine surges but also from aberrant peripheral blood volume redistribution, precipitates hypoperfusion and reduced vascular tone (86, 87).

In septic shock, the body initially releases large amounts of endogenous catecholamines for compensation. However, sustained high levels of stimulation lead to adrenergic receptor dysfunction, with the core mechanisms involving receptor downregulation, activation of G protein-coupled receptor kinases (GRK) resulting in receptor phosphorylation and uncoupling from G proteins, as well as reduced responsiveness of downstream signaling pathways (88). This desensitization is specifically manifested as: β1-adrenergic receptor dysfunction causes a significant decrease in the heart’s responsiveness to catecholamines, leading to reduced myocardial contractility and decreased cardiac output, which is the core of sepsis-induced myocardial dysfunction (SIMD); meanwhile, α1-adrenergic receptor dysfunction results in a weakened vascular response to vasoconstrictors, causing refractory hypotension and vascular paralysis. The myocardial suppression induced by β1-receptor desensitization and the vascular paralysis induced by α1-receptor desensitization act together, exacerbating the vicious cycle of septic shock and are closely associated with increased mortality (89).

### 2.5 Complement system activation

**Figure 5:**
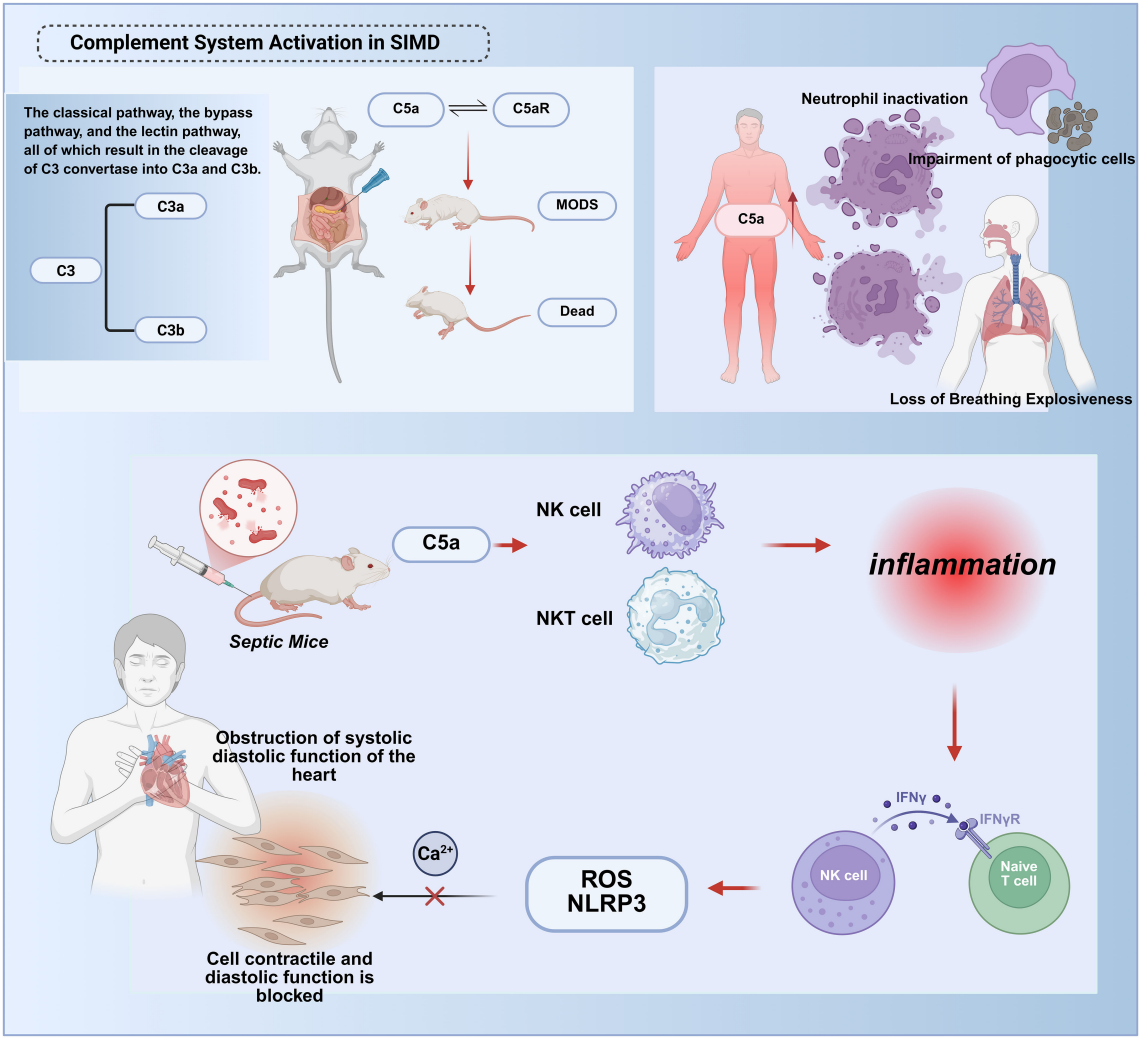
Molecular mechanisms of complement system hyperactivation in septic cardiac injury. The complement system exerts dual roles in sepsis. During early stages, activation via the classical, alternative, or lectin pathways cleaves C3 into C3a/C3b, facilitating pathogen clearance and immune regulation. However, excessive late-phase activation drives systemic inflammation and tissue injury.In early sepsis, the complement activation product C5a binds to the C5a receptor (C5aR), Myocardial pyroptosis and dysfunction:C5a activates the reactive oxygen species/NOD-like receptor protein 3 (ROS/NLRP3) inflammasome pathway, inducing cardiomyocyte pyroptosis;Disrupted intracellular calcium homeostasis impairs myocardial contraction/relaxation.Immune dysregulation:C5a activates natural killer (NK) and NKT cells, amplifying cytokine storms (e.g., TNF-γ release) and recruiting immune cells to infection sites.These synergistic mechanisms culminate in irreversible myocardial damage and cardiac failure. This figure was created using BioRender (biorender.com).

The complement system, a critical enzymatic cascade within innate immunity, orchestrates complex molecular interactions to regulate and amplify immune responses against pathogenic invasion and aberrant cellular proliferation. This system exhibits a paradoxical role in host defense: while physiologically protective, its dysregulation or hyperactivation contributes to tissue injury in systemic inflammatory conditions such as trauma, sepsis, and severe COVID-19 (90). Experimental models confirm that complement activation product C5a exerts potent proinflammatory effects during early sepsis. Recent investigations in cecal ligation and puncture (CLP) rats demonstrate that C5a-C5a receptor (C5aR) interactions correlate with adverse outcomes, including multiorgan failure and mortality (91). (**Figure 5**).

Excessive C5a generation during sepsis associates with neutrophil “paralysis,” characterized by impaired respiratory burst activity and defective oxygen-dependent bactericidal pathways (92). Although C5aR’s mechanistic significance in sepsis pathophysiology remains incompletely defined, preclinical evidence suggests its involvement in coordinating innate immune responses. The complement cascade operates through three distinct initiation pathways—classical, lectin, and alternative—all converging on C3 convertase formation, which cleaves C3 into anaphylatoxin C3a and opsonin C3b to propagate terminal effector mechanisms (90). Murine studies reveal early myocardial complement activation, wherein C5a synergistically activates NK and NKT cells to mediate cytokine storm development. This process drives NKT/NK cell recruitment to infection sites and promotes TNF-γ release from NK and dendritic cells (91, 93). Mechanistically, C5a not only upregulates reactive oxygen species/NLRP3 (ROS/NLRP3) signaling to induce pyroptosis but also disrupts cytoplasmic ion channel homeostasis and intracellular calcium flux, thereby suppressing cardiomyocyte contractile and diastolic functions.

### 2.6 Extracellular vesicles

**Figure 6:**
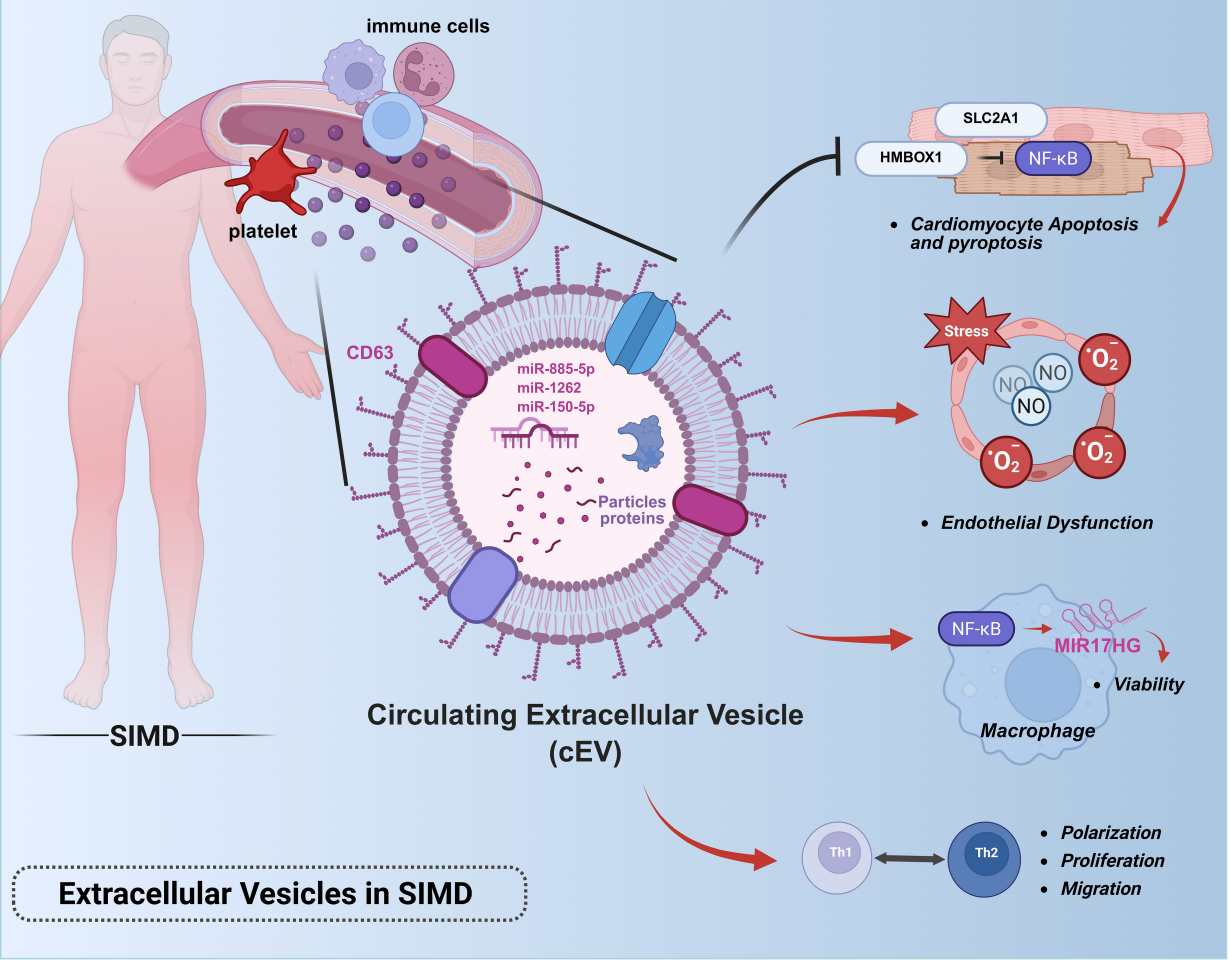
Roles of extracellular vesicles in sepsis-induced myocardial dysfunction.This figure illustrates the pathophysiological role of extracellular vesicles in sepsis-associated cardiac injury: Circulating EVs derived from immune cells (e.g., neutrophils, macrophages) propagate systemic inflammation by delivering pro-inflammatory cargo (miRNAs, cytokines, DAMPs) to cardiomyocytes and endothelial cells.Key pathways include miR-885-5p/HMBOX1/NF-κB-mediated pyroptosis, TLR4/NF-κB activation in macrophages via MIR17HG, and miR-1262/SLC2A1-driven metabolic dysregulation.Platelet-derived EVs exacerbate endothelial dysfunction through NO and superoxide overproduction.Note: This figure was created using BioRender (biorender.com).

Emerging insights into intercellular communication mechanisms have propelled EVs into scientific prominence as novel mediators of biological signaling, owing to their unique structural architecture, dimensional characteristics, and molecular inheritance from parent cells (94). Current operational definitions characterize EVs as non-replicative, lipid bilayer-enclosed structures spanning nano- to micro-scale dimensions, with particular research emphasis on small EVs (sEVs) measuring 30–150 nm. These sEV subpopulations, including exosomes (derived from multivesicular bodies) and ectosomes (formed through plasma membrane budding), constitute the primary focus of contemporary EV research (95). Comprehensive profiling reveals that virtually all mammalian cells constitutively secrete sEVs containing diverse bioactive payloads encompassing nucleic acids, proteins, and cytokines. Upon internalization by recipient cells, these bioactive cargoes evade endosomal degradation and orchestrate downstream signaling cascades through sophisticated molecular mechanisms (96–98). Recent investigations employing multi-omics approaches have uncovered the dual pathophysiological roles of EVs in sepsis-associated organ dysfunction. Paradoxically, while EVs from activated immune cells propagate inflammatory responses and exacerbate tissue damage through systemic molecular dissemination, those derived from healthy tissues – particularly stem cell populations – demonstrate remarkable therapeutic potential (99–102). This dichotomy underscores EVs’ capacity to function as molecular “ double-edged swords “ in sepsis pathophysiology, simultaneously driving pathological progression and enabling innovative therapeutic interventions. (**Figure 6**).

During the pathogenesis of sepsis, EVs exacerbate disease progression by delivering cargo components (including miRNAs, mRNAs, and bioactive proteins) to target cells such as immune cells, endothelial cells, and cardiomyocytes, thereby promoting cellular apoptosis, amplifying inflammatory cascades, and disrupting efferocytosis-mediated clearance of apoptotic cells - processes that collectively aggravate tissue necrosis (103–106). Investigations demonstrate that sepsis-derived EVs circulating in murine blood harbor substantial inflammatory mediators and chemokines. These vesicles exhibit prolonged circulatory half-lives compared to soluble cytokines, enabling sustained dynamic interactions that propagate systemic pathological responses throughout sepsis progression (107). Quantitative analyses reveal peak EV concentrations in circulation 48 hours post-sepsis induction, with functional characterization showing these vesicles enhance TH1/TH2 polarization and facilitate T-cell proliferation/migration (108). A mechanistic study employing cecal ligation and puncture-derived exosomes (CLP-exo) identified their capacity to activate the NF-κB pathway in macrophages, resulting in significant upregulation of long non-coding RNA MIR17HG. This regulatory axis critically sustains macrophage viability, as evidenced by reduced cellular vitality following MIR17HG inhibition and attenuated MIR17HG expression upon NF-κB suppression, confirming their bidirectional regulatory relationship. These findings establish circulating EVs as modulators of macrophage homeostasis in sepsis pathophysiology (105). Subsequent mechanistic studies have delineated the central role of circulatory EVs in sepsis-induced cardiac injury (109), Multiple lines of evidence indicate platelet-derived EVs mediate myocardial depression through NO and superoxide-induced endothelial dysfunction, ultimately leading to cardiomyocyte damage (110). Pharmacological inhibition of EV biogenesis via GW4869 administration effectively preserved cardiac function and attenuated myocardial inflammation in septic mice (111). Current research implicates EV-associated miRNAs as key mediators of programmed cardiomyocyte death and metabolic dysregulation in sepsis. Two clinical studies investigating serum exosomes from sepsis patients (Sepsis-exo) revealed distinct pathogenic mechanisms: 1. miR-885-5p-mediated activation of the HMBOX1/NF-κB axis triggers NLRP3/caspase-1/GSDMD-N-dependent pyroptosis, elevating IL-1β/IL-18 secretion by 2-3-fold and exacerbating cardiac injury in septic rats (112). 2. Time-dependent hsa-miR-1262 enrichment in Sepsis-exo suppresses SLC2A1 expression through 3’UTR targeting, leading to increased cardiomyocyte apoptosis and impaired glycolysis (113).

Clinical investigations have established a significant correlation between plasma extracellular vesicle levels and both organ failure severity and predictive mortality in sepsis patients, with markedly elevated EV concentrations detected in septic shock patients compared to controls (114). A subsequent miRNA sequencing study of plasma EVs from septic shock patients revealed their enrichment with biological signals associated with inflammatory responses, oxidative stress, and cell cycle regulation (115). These findings collectively demonstrate the dynamic evolution of plasma EVs during sepsis progression and their tight association with disease advancement, suggesting potential diagnostic and prognostic utility through EV monitoring. A clinical study profiling miRNA signatures in neutrophil-derived EVs from sepsis patients identified miR-150-5p as critically associated with SIMD development and severity (116). Comparative analysis revealed 38 differentially expressed miRNAs between SIMD and non-SIMD patients, with 27 miRNAs (23 upregulated, 4 downregulated) showing consistent expression patterns across cohorts. Multivariate logistic regression analysis demonstrated high predictive value for SIMD when combining miR-150-5p with NT-proBNP, LVEF, and SOFA scores. ROC curve analysis showed miR-150-5p alone achieved an AUC of 0.855 for SIMD prediction, with its reduced expression correlating with enhanced inflammatory responses and compromised cardioprotective mechanisms. While these findings nominate miR-150-5p as a potential SIMD biomarker, the study emphasizes the necessity for further mechanistic exploration.

## 3 Therapeutic strategies for sepsis-induced myocardial dysfunction

### 3.1 Anti-inflammatory therapeutics

**Figure 7:**
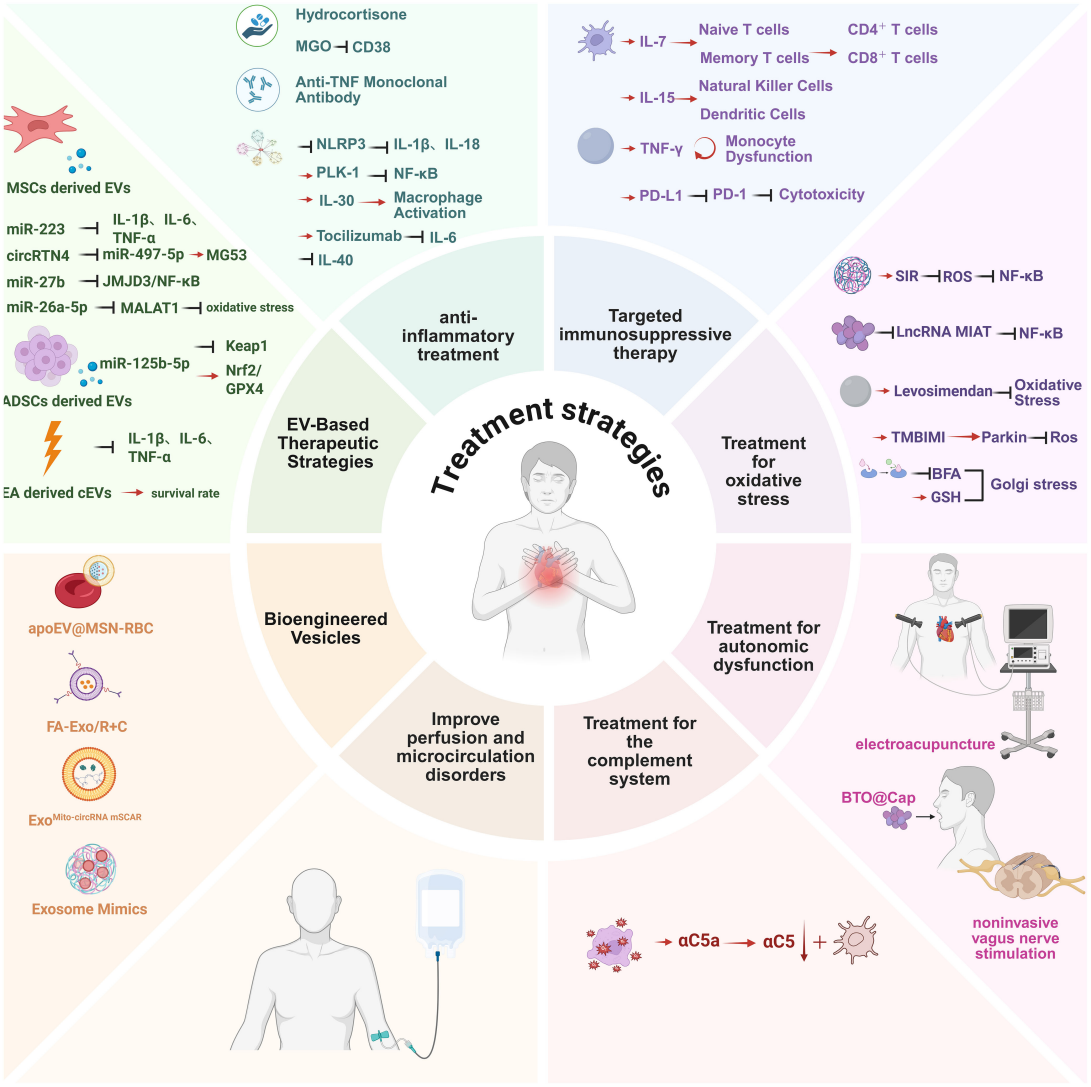
Multidimensional therapeutic strategies for sepsis-induced myocardial injury. 1. Anti-inflammatory therapeutics (e.g., NLRP3 inflammasome inhibitors, IL-6 receptor antibodies); 2. Immunomodulatory interventions (PD-1/PD-L1 checkpoint inhibitors); 3. Oxidative stress-targeted therapies (mitochondrial-targeted antioxidants, Nrf2/HO-1 pathway activation); BFA exacerbates injury, GSH rescues this effect, suggesting Golgi integrity as a therapeutic target;4. Autonomic regulation (electroacupuncture via vagal-splenic-immune reflex to suppress inflammation); Oral BTO@Cap particles combined with low-intensity US activate local vagus nerve electrical stimulation; via α7nAChR, this inhibits macrophage pro-inflammatory cytokines (TNF-α/IL-6), reducing inflammation and increasing survival by 67% in septic mice. The particles enhance gastric adhesion via TRPV1 targeting and avoid systemic absorption due to their micro-sized structure; a portable US device enables home-based therapy; 5. Complement system modulation (C5a receptor antagonists);Microcirculatory optimization (fluid resuscitation and vasoactive agents); 6. Extracellular vesicle-based therapies (stem cell-derived EVs delivering miR-223, engineered vesicles for targeted NF-κB inhibitor delivery).Arrows indicate mechanistic interactions:Red horizontal arrows denote activation or promotion.Black arrows indicate inhibition or suppression. This figure was created using BioRender (biorender.com). BFA: Golgi agonist, GSH: Golgi inhibitor.

Sepsis is pathologically defined as a systemic inflammatory response to infection (117, 118). While inflammation constitutes a localized protective mechanism against microbial invasion or tissue injury, its precise regulation is critical, as both insufficient and excessive immune responses contribute to morbidity and reduced survival (119). Contemporary research highlights immune dysregulation—characterized by hyperinflammation coupled with immunosuppression—as a widely accepted pathogenic mechanism underlying sepsis. Consequently, anti-inflammatory interventions remain pivotal in sepsis management. Accumulating evidence demonstrates that blockade of inflammatory cascades effectively mitigates sepsis-induced cardiomyocyte pyroptosis, including inhibition of TNF, IL-1, IL-6, IL-7, IL-15, coagulation factors, and complement C5a (120, 121). (**Figure 7**).

Notably, activation of NLRP3 inflammasome plays a central role in SIMD by driving IL-1β and IL-18 secretion (122). Pharmacological suppression of NLRP3 signaling or downstream IL-1β-mediated cytokine responses has long been proposed as a therapeutic strategy for inflammatory disorders. Given the established correlation between excessive inflammation and SIMD pathophysiology, targeting NLRP3 inflammasome activation represents a promising therapeutic avenue (123).

The canonical NF-κB inflammatory pathway has been implicated as a major contributor to SIMD (124). Experimental studies reveal that Polo-like kinase 1 (Plk-1) acts as an inhibitory kinase for IκB kinase α (IKKα). Plk-1 inhibition attenuates NF-κB pathway activation in LPS-challenged murine hearts and neonatal rat cardiomyocytes (NRCMs) (125). The lncRNA MCM3AP-AS1 attenuates myocardial injury by sponging miR-501-3p to upregulate CADM1, thereby inhibiting STAT3/NF-κB-driven inflammation and oxidative stress, with overexpression improving survival, cardiac function, and mitochondrial integrity in sepsis models (126). Nevertheless, the complex immunopathogenesis of sepsis hampers the development of precision immunotherapies. Current targeted anti-inflammatory regimens remain suboptimal, compounded by the high heterogeneity of inflammatory responses across disease stages and individuals. Clinically, empirical approaches—including blood culture-guided broad-spectrum antibiotics—predominate. Hence, advancing research on early-stage targeted pharmacotherapy to arrest disease progression before multiorgan failure warrants prioritized investigation.

To date, the Surviving Sepsis Campaign guidelines recommend short-course hydrocortisone (200–300 mg/day for ≤7 days or until vasopressor independence) as the sole adjunctive therapy for refractory septic shock (127).Preclinical studies suggest that early anti-TNF monoclonal antibody administration prevents lethal tissue damage during bacterial invasion, while delayed ethyl pyruvate-mediated reduction of serum HMGB1 concentrations rescues animals from sepsis-induced mortality.

Emerging evidence positions IL-6 as a robust biomarker for sepsis severity and prognosis (128–133). Prolonged IL-6 elevation in septic patients implies a broader therapeutic window for IL-6 blockade compared to other cytokines, positioning it as a novel strategy for severe sepsis and septic shock (134). Mechanistically, IL-30—a novel IL-6 family cytokine—demonstrates cardioprotective effects in sepsis by suppressing proinflammatory macrophage polarization and pyroptosis (135). Clinical trials of tocilizumab, a humanized anti-IL-6 receptor antibody, show efficacy in cytokine release syndromes, with optimal responses observed in patients exhibiting elevated IL-6, IFN-γ, IL-8, IL-10, and MCP-1 levels (134). Critical considerations for anti-inflammatory agents include: (1) restricted application to patients with marked cytokine elevation; (2) time-sensitive administration during early sepsis; and (3) transient therapeutic effects. Integrated analysis of multidimensional data underscores that dual modulation of inflammatory mediators and associated signaling pathways represents a cornerstone in SIMD management.

A recent study has identified a unique subset of cardiac-resident macrophages (MacL) characterized by CD163^+^RETNLA^+^ expression that confers cardio-protection in SIMD through TREM2-dependent mechanisms (136). Integrated single-cell RNA sequencing and fate-mapping analyses revealed that MacL exhibits self-renewal capacity and transcriptional enrichment in endocytosis-related pathways, enabling active clearance of dysfunctional mitochondria expelled by cardiomyocytes to maintain cardiac homeostasis. Notably, genetic ablation of TREM2 markedly attenuated MacL proliferation and mitochondrial scavenging capacity, resulting in extracellular mitochondrial accumulation, exacerbated inflammatory responses (elevated TNF-α and IL-1β levels), deteriorated cardiac function, and reduced survival in murine models. Strikingly, intrapericardial transplantation of TREM2-overexpressing MacL cells reversed these pathological phenotypes, restoring mitochondrial integrity and suppressing systemic inflammation. These findings establish MacL as a critical immune-metabolic regulator in SIMD.

### 3.2 Immunomodulatory therapeutics

Historically, most pharmacotherapeutic trials in sepsis have focused on attenuating pathogen-induced hyperinflammatory responses during early disease stages by neutralizing inflammatory mediators. However, a substantial proportion of patients receiving broad-spectrum antimicrobial therapy fail to achieve clinical resolution and subsequently develop nosocomial infections, underscoring limitations of this unidimensional approach (58, 137). Consequently, immunostimulatory strategies to augment host immunity have emerged as critical interventions for sepsis patients in the immunosuppressive phase, addressing both primary pathogen clearance and secondary infection prevention (61).

Contemporary immunotherapy paradigms emphasize precision-targeted, temporally calibrated interventions. Emerging evidence from clinical trials highlights the therapeutic potential of GM-CSF, a cytokine governing neutrophil/monocyte activation and macrophage differentiation, in reversing sepsis-associated immunosuppression (138). Mechanistically, IL-7 drives lymphocyte repopulation by promoting naive and memory T-cell proliferation, effectively doubling circulating CD4^+^/CD8^+^ T-cell counts and increasing splenic/lymph node cellularity (139). This cytokine-mediated amelioration of lymphopenia and T-cell exhaustion demonstrates prognostic significance in septic hosts. Parallel studies reveal interferon-γ (IFN-γ) restores monocyte functionality, while interleukin-15 (IL-15) exhibits unique immunostimulatory properties through natural killer cell activation and dendritic cell expansion—cell populations critically depleted during sepsis pathogenesis.

Biomarker-Guided Therapeutic Stratification: The pathophysiological heterogeneity of sepsis necessitates precise immunological phenotyping to differentiate hyperinflammatory versus immunosuppressive states, as misaligned immunomodulation may exacerbate clinical outcomes (54). Biomarker-driven stratification enables phase-specific therapeutic optimization. Cardiac-specific biomarkers previously validated in sepsis research now inform novel targeting strategies, including programmed cell death protein 1 (PD-1) and cytotoxic T-lymphocyte-associated protein 4 (CTLA-4) checkpoint inhibition. PD-1 signaling suppresses T-cell proliferative capacity, cytokine production, and cytotoxic activity—a mechanism exploited during chronic viral infections (e.g., HIV-1, hepatitis) through sustained antigen exposure and consequent T-cell exhaustion (140, 141). Clinical investigations demonstrate that PD-1/PD-L1 axis blockade reverses this immunosuppressive phenotype, restoring pathogen clearance capacity. This multimodal framework—integrating immune reconstitution, temporal biomarker profiling, and checkpoint modulation—represents a paradigm shift in sepsis management, addressing the dual challenges of pathogen eradication and immune disorder.

### 3.3 Oxidative stress-targeted therapeutics

Emerging evidence from murine sepsis models demonstrates that mitochondrial-targeted interventions improve cardiac functional recovery and reduce mortality, consistent with the critical role of mitochondrial dysmetabolism in SIMD (71). Ultrastructural and functional mitochondrial abnormalities manifest early in sepsis pathogenesis, where ROS overproduction synergizes with calcium overload to trigger mPTP opening, facilitating extramitochondrial release of mtDNA fragments. These mtDNA damage-associated molecular patterns (mtDNA DAMPs) activate innate immune pathways recognized as pivotal contributors to intramyocardial inflammation (10).

Notably, pharmacological activation of sigma-1 receptor (S1R) mitigates mitochondrial oxidative stress via the Nrf2/HO-1 signaling axis, attenuating sepsis-induced cardiomyocyte apoptosis and preserving cardiac performance. S1R activation promotes nuclear translocation of Nrf2, inducing downstream antioxidant enzymes (e.g., HO-1) to counteract oxidative damage (142). Complementary research by Yue Peng et al. reveals IL-6 exerts cardioprotective effects against oxidative stress during early LPS-induced sepsis. Il-6-deficient mice exhibited compromised cardiac rhythm following LPS challenge, whereas IL-6 supplementation alleviated oxidative injury. Mechanistically, IL-6 enhances Nrf2 nuclear translocation and antioxidant gene expression, maintaining cardiomyocyte redox homeostasis. Nrf2 ablation abolished IL-6-mediated protection, confirming this pathway’s indispensability (143).

Preclinical studies further identify levosimendan as a modulator of mitochondrial quality control, activating PINK1-Parkin-mediated mitophagy and suppressing Drp1-dependent mitochondrial fission through JNK-LATS2 signaling. This dual mechanism preserves mitochondrial integrity, mitigating oxidative stress and cardiac dysfunction in septic models (144) (145).

Intriguingly, Peng-Cheng Xing et al. delineate a pro-oxidative role for long non-coding RNA MIAT in septic cardiomyopathy, wherein its overexpression exacerbates inflammation and oxidative stress via miR-330-5p/TRAF6/NF-κB axis dysregulation (146). Collectively, these findings underscore the therapeutic potential of early-phase oxidative stress modulation in preserving myocardial integrity and improving SIMD outcomes. Targeted strategies—ranging from mitochondrial stabilization to redox signaling modulation—represent promising frontiers in sepsis-associated cardiac injury management.

### 3.4 Therapeutics targeting autonomic nervous system dysregulation

Dysregulation of the ANS, a critical mediator of host responses to infection, has been implicated in sepsis-associated organ dysfunction (84). Accumulating evidence suggests acupuncture modulates sympathetic hyperactivity and attenuates maladaptive cardiac remodeling, including pathological hypertrophy (147, 148). Modern mechanistic studies further reveal that acupuncture exerts systemic anti-inflammatory effects by rebalancing pro-/anti-inflammatory cytokines through dual modulation of vagal and sympathetic pathways, thereby preventing progression to cytokine storms (119). This therapeutic action is mediated via somato-autonomic-immune reflexes, including somato-sympathetic-splenic, somato-sympathetic-adrenal, somato-vagal-splenic, and somato-vagal-adrenal neural circuits (149). Data on electroacupuncture and vagus nerve stimulation primarily come from preclinical studies or small-scale clinical trials.

The discovery that cholinergic neurons reflexively inhibit acute inflammation has revolutionized our understanding of neuroimmune regulation, analogous to autonomic control of vital physiological functions (119). Electroacupuncture preconditioning may activate the sympathetic nervous system, inducing β-adrenergic receptor desensitization through a mechanism similar to ischemic preconditioning, thereby exerting cardioprotective effects. However, whether electroacupuncture alleviates myocardial ischemia-reperfusion injury (MIRI) by modulating autonomic nervous activity, and its specific mechanisms, remain to be elucidated. The study by Zhang et al. suggests that the neuro-immune regulatory pathway of electroacupuncture is intensity-dependent: high-intensity electroacupuncture (3.0 mA) can activate the spinal sympathetic-splenic pathway, enhancing the role of α2ARs, and this effect can be blocked by yohimbine (an α2ARs antagonist) or splenectomy; conversely, weak stimulation at 0.5 mA, although insufficient to activate this pathway, can initiate the vagal-splenic pathway to produce anti-inflammatory effects. These findings not only reveal the complexity of electroacupuncture in regulating immunity through different neural pathways, but also further highlight the critical role of adrenergic receptor dysfunction in the development and progression of severe conditions such as sepsis and shock (149). This paradigm shift opens avenues for modulating inflammatory diseases through selective neuromodulation of “hard-wired” neural circuits. Experimental studies by Zhiyang Wu et al. demonstrate that electroacupuncture (EA) at PC6 (Neiguan) improves sepsis-induced left ventricular dysfunction, suppresses proinflammatory cytokine overproduction, and reduces inflammatory cell infiltration—effects dependent on intact vagal signaling. Complementary clinical investigations by Sun F.Y. et al. show that EA combined with standard care enhances left ventricular ejection fraction, reduces circulating caspase-3, Bcl-2, and troponin levels, and mitigates cardiomyocyte apoptosis in septic patients (150). Preclinical models elucidate EA’s cardioprotective mechanisms: (1) Downregulation of myocardial TNF-α, IL-6, malondialdehyde (MDA), and myeloperoxidase (MPO); (2) Enhancement of SOD activity to suppress oxygen radical release; (3) Macrophage polarization from proinflammatory M1 to anti-inflammatory M2 phenotypes, accompanied by reduced TNF-α/IL-1β/IL-6 and elevated IL-10 expression. Clinically translatable approaches include pharmacological or electrical vagus nerve stimulation to modulate cytokine release—a strategy with therapeutic relevance across inflammatory disorders (151). Recent advances have highlighted a novel approach to modulate the cholinergic anti-inflammatory pathway (CAIP)—a crucial component of the body’s innate defense system that finely coordinates responses to tissue injury or infection. This method utilizes non-invasive focused ultrasound (US) to perform noninvasive vagus nerve stimulation (VNS), which acts through the splenic vagus nerve to activate the CAIP, thereby alleviating endotoxin-induced cytokine production (152). A promising study demonstrated that orally ingestible piezoelectric particles (BTO@Cap), activated by low-intensity pulsed ultrasound (US), generate localized electrical pulses that selectively stimulate vagal afferent fibers. This stimulation triggers CAIP activation, suppressing pro-inflammatory cytokine release (e.g., TNF-α, IL-6) via α7 nicotinic acetylcholine receptor (α7 nAChR)-mediated macrophage modulation. In murine sepsis models, this approach reduced systemic inflammation, attenuated myocardial injury, and improved survival by 67%, with minimal thermal impact due to optimized US parameters (0.3 W/cm²). The particles’ TRPV1 receptor-targeting capability ensured gastric adherence, while their microscale size prevented systemic absorption, enhancing biosafety. Complementing traditional interventions like electroacupuncture, which improves cardiac function via vagal-splenic-immune reflexes, this noninvasive VNS strategy offers advantages in clinical translation. Portable US devices enable home-based therapy, addressing accessibility challenges in critical care settings.

Statins demonstrate pleiotropic benefits in sepsis management by reducing sympathetic outflow and preserving parasympathetic tone, which correlates with attenuated systemic inflammation and lower sepsis incidence in adults (153, 154). Collectively, these findings establish ANS-targeted interventions as multifaceted strategies that address both neuroautonomic imbalance and subsequent inflammatory cascades in septic cardiomyopathy. Studies suggest that electrical stimulation, in particular, serves as an adjunctive rather than a replacement therapy for standard sepsis care. It is important to note, however, that the current evidence is largely derived from preclinical animal models and small-scale clinical studies. Therefore, these promising findings remain pending validation through large-scale, rigorous randomized controlled trials in sepsis patients.

### 3.5 Complement system-targeted therapeutics

The complement system functions as a first-line danger sensor, with its activation serving as an upstream driver of inflammatory cascades. Elevated levels of the anaphylatoxin C5a—a key complement activation product—exacerbate systemic inflammation during sepsis. Experimental studies demonstrate that αC5a receptor blockade enhances neutrophil binding capacity and significantly improves survival rates in septic murine models, offering novel therapeutic perspectives for clinical translation (155). Despite sepsis’s pathophysiological complexity, complement inhibition monotherapy has shown partial efficacy in mitigating trauma-associated sepsis, underscoring its modulatory potential (90). Critically, complement activation exhibits a threshold-dependent effect in severe inflammatory models: once systemic complement accumulation surpasses a critical threshold, patients may progress beyond the optimal therapeutic window, emphasizing the importance of early intervention timing.

### 3.6 Microcirculatory optimization and perfusion enhancement

Sepsis-induced inflammatory responses precipitate microvascular dysfunction, exacerbating myocardial ischemia-hypoxia and compounding cardiac injury (156). As microcirculatory restoration represents the ultimate objective of resuscitation, achieving normative microvascular perfusion is prioritized in septic shock management (157). Early-phase sympathetic overactivation preferentially compromises cutaneous microcirculation, necessitating prompt fluid resuscitation to ameliorate tissue hypoxia (158). Current guidelines emphasize comprehensive hemodynamic assessment during initial septic shock evaluation to guide tailored resuscitation strategies.

The absence of standardized therapies for microcirculatory dysfunction—a pathophysiological cornerstone of sepsis progression—highlights the clinical reliance on supportive care. Emerging evidence positions microcirculatory preservation as a critical therapeutic target, requiring multimodal approaches to address endothelial dysfunction, capillary leakage, and impaired oxygen extraction (159). While early fluid administration remains fundamental, advanced strategies integrating vasoactive agents, anti-inflammatory therapies, and mitochondrial protection are under investigation to break the vicious cycle of hypoperfusion and cellular energy failure (159).

This dual focus on complement modulation and microcirculatory optimization reflects the evolving paradigm in sepsis management—transitioning from global hemodynamic targets to precision interventions addressing molecular drivers and end-organ perfusion deficits.

### 3.7 Extracellular vesicle-based and engineered vesicle therapeutic strategies

#### 3.7.1 Stem cell-derived extracellular vesicle

Recent advances have elucidated the therapeutic mechanisms of stem cell-derived extracellular vesicles in SIMD, primarily mediated through their cargo of functional nucleic acids that regulate inflammatory, oxidative stress, and cell death pathways. Studies demonstrate that exosomes from mesenchymal stem cells (MSCs) significantly improve cardiac function and survival rates in septic mice via miR-223 delivery, which suppresses pro-inflammatory cytokine release (TNF-α, IL-1β, IL-6) from macrophages and attenuates cardiomyocyte apoptosis by targeting Sema3A and Stat3. Notably, miR-223-depleted MSCs lose this protective efficacy (160). Furthermore, MSC-derived exosomes deliver circular RNA circRTN4, which activates the MG53 pathway by sponging miR-497-5p, reducing myocardial reactive oxygen species accumulation and apoptosis. Separately, pyroptotic MSCs generate specialized vesicles (pyroEVs) that reduce sepsis mortality by preserving B-cell populations (161, 162). Additional studies reveal that exosomal miR-27b suppresses inflammatory cytokine release via JMJD3/NF-κB axis inhibition (163), miR-26a-5p mitigates oxidative stress by targeting MALAT1 (164), and adipose-derived stem cell (ADSC) exosomes inhibit pulmonary endothelial cell ferroptosis through the miR-125b-5p/Keap1/Nrf2/GPX4 pathway (165). While optimization of dosing regimens (e.g., single MSC-EV administration in ovine sepsis models (166)) may enhance therapeutic outcomes, current evidence collectively supports that stem cell EVs exert cardioprotective and multi-organ protective effects via multi-target regulatory networks. These findings not only delineate core molecular mechanisms underlying EV-based sepsis therapy but also provide a theoretical foundation for developing cell-free treatment strategies. EVs exhibit substantial heterogeneity, with distinct cellular origins conferring divergent biological effects. For instance, combined administration of MSC- and hepatocyte-derived exosomes with imipenem synergistically suppresses systemic and hepatic inflammation in septic mice (significantly reducing pro-inflammatory cytokines, hepatic enzymes, bacterial load, while elevating anti-inflammatory cytokines and survival rates). Mechanistically, MSC exosomes enhance systemic immunomodulation (via Treg expansion), whereas hepatocyte exosomes preferentially repair hepatic damage, jointly maintaining immune homeostasis (167).

#### 3.7.2 Electroacupuncture-driven circulating extracellular vesicles

A novel study demonstrated that EA-induced endogenous circulating serum extracellular vesicles (Acu-exo) and their miRNA cargo significantly improve survival rates and mitigate inflammatory responses in septic mice (168). EA increased the 7-day survival rate from 26.67% to 66.70% (P < 0.05), concurrently reducing serum TNF-α and IL-6 levels and alleviating pulmonary edema (evidenced by decreased wet-to-dry weight ratios). Intraperitoneal administration of Acu-exo recapitulated EA’s therapeutic efficacy, with *in vivo* imaging confirming their targeted pulmonary accumulation. Mechanistically, Acu-exo suppressed macrophage-derived pro-inflammatory cytokines (TNF-α, IL-6, IL-1β) and carried 53 differentially expressed miRNAs (40 upregulated, 13 downregulated). These miRNAs modulate multi-target networks, including MAPK and PI3K-Akt signaling pathways, with key involvement of HSP90AA1, GRB2, and EGFR. This work elucidates EA’s capacity to orchestrate a multi-target regulatory network via circulating EVs, proposing a promising strategy for sepsis therapy.

#### 3.7.3 Bioengineered vesicles

Bioengineered extracellular vesicle-based nanoplatforms, leveraging their high biocompatibility, low immunogenicity, and engineerability, enable organ-specific targeted therapy while enhancing therapeutic efficacy (169). Emerging studies demonstrate that engineered vesicles mitigate sepsis-associated cardiac injury and multi-organ dysfunction through diverse mechanisms (170). Zheng et al. (171) developed folate-functionalized vesicles co-loaded with resveratrol and celastrol (FA-Exo/R+C), which fully restored cardiac ejection fraction and improved hepatic/renal parameters in septic mice by suppressing systemic inflammation and modulating NF-κB/ERK pathways. Another study employed engineered exosomes (Exo-srκB) to deliver NF-κB super-repressors, significantly enhancing survival rates and attenuating acute kidney injury in septic mice (172). Furthermore, erythrocyte-driven engineered apoptotic vesicles (apoEV@MSN-RBC) integrating iron chelation, toxin neutralization, and anti-inflammatory miRNA delivery increased survival rates in hyperferric sepsis models (173). To address mitochondrial dysfunction, a mitochondrial-targeted circRNA mSCAR delivery system (Exo[Mito]) restored myocardial function and reduced inflammatory cytokine expression by regulating mitochondrial reactive oxygen species and macrophage polarization (174). Notably, induced exosomes (iExo) secreted by LPS-stimulated melanoma cells, combined with hyaluronic acid-polyethyleneimine (HA-PEI)-based biomimetic nanoparticles (CII) delivering miRNA cocktails, suppressed TLR4/NF-κB signaling, improving survival and alleviating organ pathology in animal models (175). Collectively, these studies establish that engineered EVs reverse sepsis-induced cardiac dysfunction and systemically protect multiple organs through precision delivery, multi-target regulation, and synergistic mechanisms, offering a high-efficacy, low-toxicity therapeutic paradigm for clinical translation.

In summary, from anti-inflammatory and immunomodulatory approaches to targeting organelles and neuroimmune interactions, therapeutic strategies for SIMD demonstrate a trend towards multi-level and multi-target interventions. To systematically synthesize the aforementioned therapeutic strategies with their corresponding pathophysiological foundations, **Table 2** below summarizes the key pathological processes, core targets, and respective therapeutic strategies for septic cardiomyopathy, clearly outlining the translational pathway from mechanistic exploration to clinical intervention.

**Table 2:** Potential therapeutic targets and strategies for septic cardiomyopathy.

Septic cardiomyopathy: pathophysiological processes and therapeutic targets					
Pathophysiological process	Involved cell types /organelle dysfunction	Key receptors	Therapeutic targets	Trial phase	References
Inflammatory Response	Pro-inflammatory cytokine release(Immune Cells:macrophages, neutrophils,Cardiomyocytes: pyroptosis, apoptosis, NLRP3 Inflammasome activation)	TLR4 (LPS recognition), NF-κB, IL-40	Anti-cytokine Therapy (e.g., Tocilizumab), NLRP3 Inhibitors, IL-40 Inhibition	Preclinical: NLRP3 Inhibitors, IL-40 Inhibition Clinical: Tocilizumab	(13, 15, 16)
Immunosuppression	Lymphocyte exhaustion, immune disorder:T Lymphocytes (CD4^+^, CD8_+_ exhaustion, apoptosis), Monocytes/Macrophages (dysfunction)	PD-1/PD-L1, IL-10	Immune Checkpoint Inhibitors (e.g., anti-PD-L1), Immunostimulants (e.g., IL-7, GM-CSF)	Clinical	(55, 57, 95, 176)
Calcium Dyshomeostasis & Cell Death	Sarcoplasmic Reticulum (SERCA2a dysfunction), Endoplasmic Reticulum (IP3R2-mediated Ca²^+^ release), Cardiomyocytes (apoptosis, pyroptosis, ferroptosis)	SERCA2a, IP3R2, GSDMD	SERCA2a Stabilizers (e.g., PKM2 activation, SIRT2-mediated desuccinylation), IP3R2 Inhibitors, Anti-apoptotic/pyroptotic Agents	Preclinical	(39–41, 112)
Oxidative stress	Cardiomyocytes, macrophages (mPTP opening, mitochondrial swelling)	NLRP3, Nrf2/HO-1 Pathway	Telmisartan, Statins, Mitochondria-targeted Antioxidants (e.g., CoQ10)	Clinical	(77, 142, 143)
Adrenergic dysfunction	Sympathetic terminals, cardiomyocytes (receptor desensitization, signaling disruption), Impaired Cholinergic Anti-inflammatory Pathway	β1, α1	Vagal stimulation, Electroacupuncture	Preclinical	(89)
Complement System Activation	Enhanced inflammation & tissue injury:Cardiomyocytes (dysfunction), Immune Cells (NK, NKT cell activation)	C5a-C5aR Axis	C5aR Antagonists	Preclinical	(91)
EV-mediated pyroptosis/therapy	Neutrophils, endothelial cells (release/uptake of EVs, inflammasome activation)	HMBOX1/NF-κB	Engineered EVs	Preclinical	(105, 111, 112)

The bolded sections in items 2 and 3 of the table represent the titles, table headers, and the clinical phases of the specified evidence, and have no special significance.

## 4 Translational research in sepsis-induced myocardial dysfunction: current landscape and future direction

Cardiac dysfunction, a critical determinant of outcomes in sepsis-induced systemic inflammation and multiorgan failure, remains a therapeutic challenge. Current anti-inflammatory strategies—including tocilizumab and corticosteroids—are constrained by narrow therapeutic windows (requiring early intervention), high interpatient heterogeneity (necessitating biomarker stratification), and insufficient targeting (e.g., broad-spectrum antibiotics’ inability to modulate immunity precisely). The central dilemma in sepsis immunotherapy lies in balancing anti-inflammatory and immunostimulatory interventions temporally, necessitating a paradigm shift from “one-size-fits-all” approaches to individualized, phase-specific therapies. Notably, sepsis-induced immunosuppression exhibits organ-specific patterns (e.g., more pronounced T-cell exhaustion in spleen versus lung), while immunostimulants risk triggering cytokine rebound (e.g., IFN-γ overactivation). Targeted interventions addressing these spatiotemporal dynamics could overcome current limitations, transitioning therapeutic goals from survival improvement to functional immune reconstitution. Recent translational advances have elucidated pathophysiological mechanisms and propelled novel therapies from bench to bedside, with ongoing clinical trials focusing on three pillars of septic cardiomyopathy: myocardial suppression, microcirculatory dysfunction, and metabolic dysregulation. (**Table 3**).

**Table 3:** Translational clinical research on sepsis and its cardiac injury.

Current situation and breakthrough direction			
Research focus area		Biomarkers	Trial Identifiers
Mechanism exploration and target validation	Inflammation and immune imbalances Endothelial injury and metabolic regulation Disorders of mitochondrial metabolism	KKS, PLA PTP1B ROS, mtDNA	NCT06080282 NCT06655389 NCT02295514 NCT05148117
Biomarker development	Inflammation-related markers Heart-specific markers	Progranulin Presepsin, RDW	NCT03280576 NCT03796715
Research on Treatment Interventions	Anti-inflammation and immune regulation Targeting mitochondria and metabolic regulation Novel antithrombotic strategies	rhBNP Coenzyme Q Inhibitors against PLA formation:CD62P/PSGL-1 blocking agent	NCT05111769 NCT05148117 NCT06655389

The bolded sections in items 2 and 3 of the table represent the titles, table headers, and the clinical phases of the specified evidence, and have no special significance.

### 4.1 Mechanistic insights and target validation

#### 4.1.1 Inflammatory-immune crosstalk

The kallikrein-kinin system (KKS) and platelet-leukocyte aggregates (PLAs) critically mediate SIMD. PLAs exacerbate myocardial inflammation via CD62P/PSGL-1-mediated leukocyte-platelet adhesion (**NCT06655389**), while dysregulated KKS activation correlates with impaired cardiac contractility (**NCT06080282**).

#### 4.1.2 Endothelial dysfunction and metabolic reprogramming

Protein tyrosine phosphatase 1B (PTP1B) modulates endothelial nitric oxide synthesis and insulin signaling during SIMD progression, with its expression levels strongly correlating with sepsis-induced organ failure severity (**NCT02295514**).

#### 4.1.3 Mitochondrial metabolic derangements

Mitochondrial dysfunction—characterized by ROS accumulation, calcium overload, and mtDNA extrusion—represents a core pathological feature of SIMD (**NCT051417**). Preclinical models demonstrate that mitochondrially targeted antioxidants improve cardiac function and survival, suggesting mitochondrial bioenergetic markers (e.g., mtDNA, 11DAMPs) as potential prognostic indicators.

### 4.2 Biomarker discovery

#### 4.2.1 Inflammation-related biomarkers

Progranulin, an early immune modulator, exhibits plasma concentration associations with sepsis severity and cardiac injury. Exosomal microRNA profiling further elucidates its epigenetic regulatory roles (**NCT03280576**).

#### 4.2.2 Cardiac-specific biomarkers

Presepsin (soluble CD14 subtype) shows significant elevation in SIMD patients, correlating positively with disease severity. Its combination with red cell distribution width (RDW) enhances prognostic accuracy (**NCT03796715**). Elevated heart-type fatty acid-binding protein (h-FABP) levels within 24 hours of admission serve as an early diagnostic and prognostic marker for septic cardiomyopathy (SI) (177).

#### 4.2.3 Immune feature markers

Based on the understanding of the dynamic immune disruption mechanisms in sepsis, its treatment strategies are shifting from rigid temporal staging to precision interventions based on real-time immune phenotypes. Utilizing key biomarkers such as low monocyte HLA-DR expression, persistent lymphocytopenia, and elevated PD-1/CTLA-4 for immune stratification is the core tool for selecting the correct timing and suitable patients. Accordingly, patients with immune disorder indicated by persistently low mHLA-DR expression may benefit from immunostimulatory therapies like GM-CSF or IFN-γ; for persistent lymphocytopenia caused by large-scale lymphocyte apoptosis, IL-7 therapy can effectively restore T cell count and function; and for the exhausted phenotype characterized by high PD-1/CTLA-4 expression on T cells, the use of immune checkpoint inhibitors during a defined immunosuppressive period can restore T cell function. This precision strategy is theoretically supported by the SIMMP-Sepsis model, which views sepsis as a continuous process from acute activation to persistent immune residuals. The aforementioned biomarkers serve as objective evidence for identifying this process and guiding targeted immune-restorative interventions (176).

### 4.3 Therapeutic interventions

#### 4.3.1 Anti-inflammatory and immunomodulatory agents

Recombinant human brain natriuretic peptide (rhBNP) demonstrates cardiorenal protective effects in sepsis-induced heart failure by suppressing cytokine release and improving microcirculation (**NCT05111769**).

#### 4.3.2 Mitochondrial and metabolic modulators

Coenzyme Q10 and other mitochondrial protectants are under evaluation for restoring myocardial energy metabolism (**NCT05148117**).

#### 4.3.3 Novel antithrombotic strategies

CD62P/PSGL-1 inhibitors targeting PLA formation emerge as promising anti-inflammatory therapies (**NCT06655389**).

Translational research faces three critical barriers: 1. Heterogeneity Management: Multi-omics integration (transcriptomics, metabolomics) to establish SIMD subtype classification systems. 2. Dynamic Monitoring: Development of point-of-care technologies (e.g., microfluidic chips) for real-time biomarker tracking. 3. Personalized Therapy: AI-driven models to optimize risk stratification and targeted drug selection.

Future breakthroughs will depend on closing the “mechanism-biomarker-therapy” loop, transitioning SIMD management from reactive support to precision medicine. By harmonizing mechanistic insights with clinical innovation, the field aims to redefine sepsis care through immune-metabolic reprogramming and functional recovery.

## 5 Conclusion

This review synthesizes the multifaceted pathogenesis of SIMD, emphasizing the interplay of dysregulated inflammation, immunosuppression, oxidative stress, autonomic dysregulation, and extracellular vesicle-mediated signaling. Emerging insights highlight mitochondrial dysfunction, m6A methylation-driven transcriptomic rewiring, and complement hyperactivation as pivotal contributors to cardiac injury, while novel therapeutic strategies—including NLRP3 inflammasome inhibitors, immunomodulators, engineered EVs, and neuroimmune modulation—demonstrate promise in restoring myocardial homeostasis. Despite advancements, translational challenges persist, notably heterogeneity in immune responses, dynamic biomarker profiling, and the need for temporally calibrated interventions. Future research must prioritize multi-omics integration, real-time monitoring technologies, and AI-driven therapeutic design to transition from reactive support to precision medicine. By bridging mechanistic complexity with clinical innovation, targeted therapies could shift the paradigm from survival-centric care to functional recovery, ultimately redefining outcomes in sepsis management.

## Glossary

ALDH2: Aldehyde dehydrogenase 2
ANS: autonomic nervous system
CVDs: cardiovascular diseases
CLP: cecal ligation and puncture
CTLA-4: cytotoxic T lymphocyte-associated protein 4
CAIP: cholinergic anti-inflammatory pathway
CARS: compensatory anti-inflammatory response syndrome
DAMPs: damage-associated molecular patterns
DCs: dendritic cells
EA: electroacupuncture
EVs: extracellular vesicles
GM-CSF: granulocyte-macrophage colony-stimulating factor
HMGB1: high mobility group box 1
HPA: hypothalamic-pituitary-adrenal
IL-1: interleukin-1
IL-1β: interleukin-1beta
IL-6: interleukin-6
IL-7: interleukin-7
IL-40: interleukin-40
ICUs: intensive care units
IP3R2: inositol 1,4,5-trisphosphate receptor type 2
IFN-γ: interferon-γ
IKKα: IκB kinase α
iNOS: inducible nitric oxide synthase
KKS: kallikrein-kinin system
K352: lysine 352
LPS: lipopolysaccharide
MAPK: mitogen activated protein kinase
MARS: mixed antagonist response syndrome
mtDNA: mitochondrial DNA
mPTP: mitochondrial permeability transition pore
MDA: malondialdehyde
MPO: myeloperoxidase
MGO: methylglyoxal
MSCs: mesenchymal stem cells
NLRP3: NOD-like receptor family pyrin domain-containing 3
NF-kB: nuclear factor kappa-B
NK cell: natural killer cell
NOX2: NADPH oxidase 2
NO: nitric oxide
PKM2: pyruvate kinase M2
PPARα: peroxisome proliferator-activated receptor α
PAMPs: pathogen-associated molecular patterns
PTP1B: protein tyrosine phosphatase 1B
Plk-1: Polo-like kinase 1
PD-1: death protein 1
ROS: reactive oxygen species
rhBNP: recombinant human brain natriuretic peptide
SIMD: sepsis-induced myocardial dysfunction
SIRS: systemic inflammatory response syndrome
SOD: enhancement of superoxide dismutase
TLR4: Toll-like receptor 4
TNF-α: tumor necrosis factor-alpha
VNS: vagus nerve stimulation
8-OHdG: hydroxy-2’-deoxyguanosine
NRCMs: neonatal rat cardiomyocytes
